# Characterization of *Aspergillus nidulans* TRAPPs uncovers unprecedented similarities between fungi and metazoans and reveals the modular assembly of TRAPPII

**DOI:** 10.1371/journal.pgen.1008557

**Published:** 2019-12-23

**Authors:** Mario Pinar, Ernesto Arias-Palomo, Vivian de los Ríos, Herbert N. Arst, Miguel A. Peñalva

**Affiliations:** 1 Department of Cellular and Molecular Biology, Centro de Investigaciones Biológicas CSIC, Madrid, Spain; 2 Department of Structural and Chemical Biology, Centro de Investigaciones Biológicas CSIC, Madrid, Spain; 3 Proteomics Facility, Centro de Investigaciones Biológicas CSIC, Madrid, Spain; 4 Section of Microbiology, Imperial College London, London, United Kingdom; University of Melbourne, AUSTRALIA

## Abstract

TRAnsport Protein Particle complexes (TRAPPs) are ubiquitous regulators of membrane traffic mediating nucleotide exchange on the Golgi regulatory GTPases RAB1 and RAB11. In *S*. *cerevisiae* and metazoans TRAPPs consist of two large oligomeric complexes: RAB11-activating TRAPPII and RAB1-activating TRAPPIII. These share a common core TRAPPI hetero-heptamer, absent in metazoans but detected in minor proportions in yeast, likely originating from in vitro-destabilized TRAPPII/III. Despite overall TRAPP conservation, the budding yeast genome has undergone extensive loss of genes, and lacks homologues of some metazoan TRAPP subunits. With nearly twice the total number of genes of *S*. *cerevisiae*, another ascomycete *Aspergillus nidulans* has also been used for studies on TRAPPs. We combined size-fractionation chromatography with single-step purification coupled to mass-spectrometry and negative-stain electron microscopy to establish the relative abundance, composition and architecture of *Aspergillus* TRAPPs, which consist of TRAPPII and TRAPPIII in a 2:1 proportion, plus a minor amount of TRAPPI. We show that *Aspergillus* TRAPPIII contains homologues of metazoan TRAPPC11, TRAPPC12 and TRAPPC13 subunits, absent in *S*. *cerevisiae*, and establish that these subunits are recruited to the complex by Tca17/TRAPPC2L, which itself binds to the ‘Trs33 side’ of the complex. Thus *Aspergillus* TRAPPs compositionally resemble mammalian TRAPPs to a greater extent than those in budding yeast. Exploiting the ability of constitutively-active (GEF-independent, due to accelerated GDP release) RAB1* and RAB11* alleles to rescue viability of null mutants lacking essential TRAPP subunits, we establish that the only essential role of TRAPPs is activating RAB1 and RAB11, and genetically classify each essential subunit according to their role(s) in TRAPPII (TRAPPII-specific subunits) or TRAPPII and TRAPPIII (core TRAPP subunits). Constitutively-active RAB mutant combinations allowed examination of TRAPP composition in mutants lacking essential subunits, which led to the discovery of a stable Trs120/Trs130/Trs65/Tca17 TRAPPII-specific subcomplex whose Trs20- and Trs33-dependent assembly onto core TRAPP generates TRAPPII.

## Author summary

TRAPPs govern intracellular traffic across eukaryotes, activating the Golgi GTPases RAB1 and RAB11. Other genetically tractable fungi are emerging as alternatives to baker’s yeast for cell-biological studies. We exploit *Aspergillus nidulans*, a filamentous ascomycete that has a lifestyle highly demanding for exocytosis and, that unlike baker’s yeast, has not undergone extensive gene loss. We show that fungal and metazoan TRAPPs are more similar than previously thought, after identifying three *A*. *nidulans* subunits previously believed exclusive to metazoans and demonstrating that TRAPPI is very minor, if it exists at all. Also importantly we classified, using a novel genetic approach, essential TRAPP subunits according to their role in activating RAB1 and/or RAB11, which demonstrated that the only indispensable role for TRAPPs is mediating nucleotide exchange on these GTPases and led to the discovery of a stable four-subunit subcomplex that assembles onto the also stable seven-subunit core to form the TRAPPII holocomplex.

## Introduction

**Fig 1:**
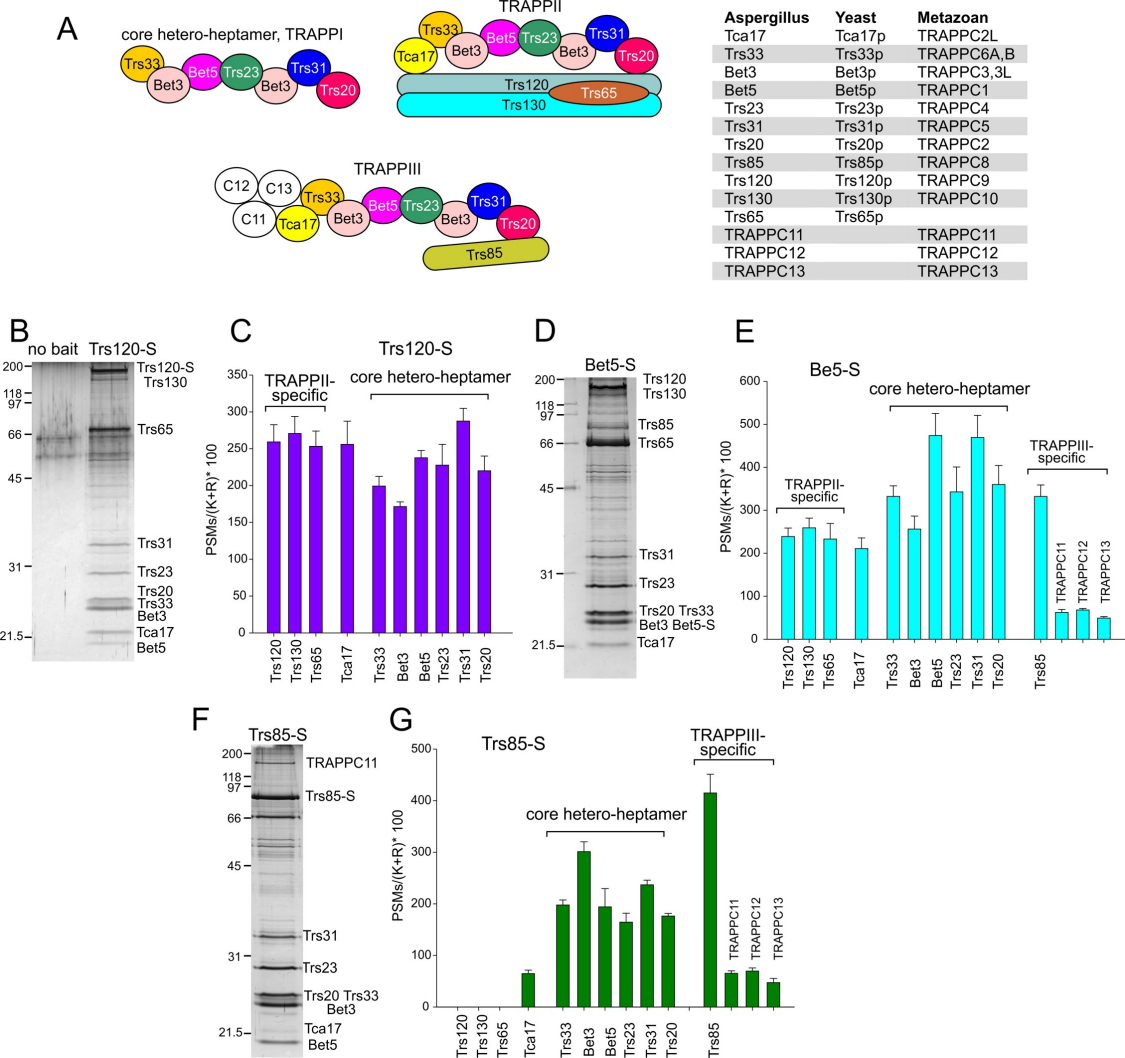
Composition of TRAPPs by shotgun proteomics. (A) Schemes depicting the subunit composition of TRAPP complexes and table showing the designation of each subunit in *A*. *nidulans*, metazoans and *S*. *cerevisiae*. To facilitate understanding we use protein designations from metazoan and yeast as appropriate throughout the text. (B) SDS-PAGE and silver staining analysis of TRAPPII purified with Trs120-S. (C) Abundance of TRAPP subunits in Trs120-S-purified material. Bars indicate spectral counts normalized to the number of Lys and Arg residues for each protein (i.e. the approximate number of tryptic peptides potentially detectable by MS/MS, multiplied by 100 and plotted as the mean +/- S.E.M. of *N =* 5 experiments). (D) SDS-PAGE of Bet5-S-purified TRAPPs. (E) Abundance of TRAPP subunits in Bet5-S-purified material (mean +/- S.E.M. of *N =* 5 experiments). (F) SDS-PAGE of Trs85-S-purified TRAPPs. (G) Abundance of TRAPP subunits in Trs85-S-purified material (mean +/- S.E.M. of *N =* 3 experiments).

TRAnsport Protein Particle (TRAPP) complexes regulate various steps of membrane traffic by promoting membrane tethering [1–3] and mediating GDP exchange on the RAB GTPases RAB1 and RAB11 [4–12]. All TRAPPs contain a ‘core hetero-heptamer’, designated TRAPPI in the ascomycete *Saccharomyces cerevisiae*, consisting of two copies of Bet3 and one copy each of Trs33, Bet5, Trs23, Trs31 and Trs20 (Fig 1A), with Bet3/Bet5/Trs23/Trs31 being minimally required to activate RABs [6]. Addition of Trs120, Trs130, Trs65 and Tca17 to the yeast ‘core hetero-heptamer’ generates TRAPPII [9,13,14], whereas addition of Trs85 generates TRAPPIII [15] (Fig 1A). These compositional changes are physiologically crucial because they shift the specificity of the GEF between RAB1 (TRAPPI and TRAPPIII) and RAB11 (TRAPPII).

The filamentous ascomycete *Aspergillus nidulans* is genetically manipulatable and its long (>100 μm) hyphal tip cells are excellent for cell biological studies [16]. Intracellular transport involving cooperation between actin- and microtubule-based motors [17–20] resembles that of metazoan cells to a greater extent than that of *S*. *cerevisiae*. *A*. *nidulans* has been used to study the maturation of *trans-*Golgi (TGN) cisternae into secretory vesicles (SVs) that are transported to the growing apex, where they accumulate until fusing with the plasma membrane, forming a fungal-specific structure denoted Spitzenkörper (SPK) [21–23]. The key driver of this maturation is RAB11 (also denoted RabE in *A*. *nidulans* [23]), whose recruitment to TGN cisternae dictates their transition towards SVs with post-Golgi identity that engage motors for transport to the SPK [17]. In *A*. *nidulans* and *S*. *cerevisiae*, activation of RAB11 (denoted Ypt31/Ypt32 in the latter) at the TGN is mediated by TRAPPII [5,7,8]. Point mutations in RAB11 facilitating spontaneous release of GDP rescue the lethality resulting from ablation of TRAPPII (the RAB11 GEF), but not of core TRAPP/TRAPPI, due to the essential requirement of the latter to activate RAB1 (RabO in *A*. *nidulans* [24]; Ypt1 in *S*. *cerevisiae* [25]).

The genome of *S*. *cerevisiae* has been shaped by reductive evolution, which has resulted in extensive gene loss and associated metabolic traits [26]. Despite the whole genome duplication that affected *S*. *cerevisiae*, this yeast has only ~5,900 coding genes in its ~12 Mbp genome, compared to the ~11,000 genes encoded in the ~30 Mbp *A*. *nidulans* genome. Although TRAPPs are strongly conserved between *S*. *cerevisiae* and metazoans [27], there appear to be two substantial differences. Firstly, *S*. *cerevisiae* TRAPPII contains a subunit, Trs65, which is absent from metazoans; And secondly, *S*. *cerevisiae* TRAPPIII lacks three subunits, TRAPPC11/TRAPPC12/TRAPPC13, present in its metazoan counterparts. Whether these differences underlie fundamental mechanistic specializations distinguishing fungal from metazoan TRAPPs or results from reductive evolution operating in the *S*. *cerevisiae* lineage remains to be determined.

Metazoan TRAPPs consist solely of TRAPPII and TRAPPIII [11], and thus it was previously thought that fungi and metazoans additionally differed in that the former would also contain TRAPPI, which would activate RAB1 for exocytosis, whereas TRAPPIII would be restricted to activating RAB1 during autophagy. However, a recent report concluded that *S*. *cerevisiae* TRAPPI is actually an *in vitro* artifact resulting from the intrinsic instability of the complexes and that it is TRAPPIII that activates RAB1 both for exocytosis and for autophagy [9]. Whether this represents a yeast-specific adaptation or whether other ascomycetes also resemble metazoans in that they also lack TRAPPI is still unknown.

Given that TRAPPII and TRAPPIII share a common core complex onto which specific subunits are added, it has been challenging to demonstrate genetically with which of the two complexes do some of the essential subunits play their role(s). Moreover, even though TRAPPs are demonstrated RAB GEFs, they have also been regarded as membrane tethers. However, their tethering role has been disputed [28] and current views deem possible that TRAPPs are more likely to act in membrane fusion beyond the actual tethering step [29].

Here we determine the relative abundance, composition and, by negative-stain electron microscopy, the overall organization, of *A*. *nidulans* TRAPP complexes. We show that *A*. *nidulans* has homologues of metazoan TRAPPC11, TRAPPC12 and TRAPPC13, which are absent from *S*. *cerevisiae*. These subunits are components of TRAPPIII, recruited to the complex by Tca17. Therefore, *A*. *nidulans* TRAPPs resemble those of metazoans to a greater extent than those of the budding yeast. By a novel lethality-rescue approach using activated RAB1 and RAB11 alleles, we establish the specific roles of core TRAPP and TRAPPII-specific subunits in RAB1 and RAB11 activation, and provide genetic evidence that the only essential role of TRAPPs is mediating nucleotide exchange on RAB1 and RAB11. By exploiting combinations of RAB1 and RAB11 mutations with ablation of TRAPP subunits we identify a stable TRAPPII-specific subcomplex consisting of Trs120, Trs130, Trs65 and Tca17, whose assembly onto the core TRAPP requires Trs20 and Trs33.

## Results

### Composition of *A*. *nidulans* TRAPPs

To investigate the composition of the different TRAPPs we purified them by S-tag single-step affinity chromatography [7]. For each genetic condition (see also below) we run two parallel purifications, using S-tagged Trs120 (a TRAPPII-specific subunit) and S-tagged Bet5 (a subunit shared by all TRAPPs) as baits. Purified complexes were analyzed by shotgun MS/MS sequencing. Fig 1B and 1D show SDS-PAGE analyses of complexes purified with Trs120-S and Bet5-S, respectively, whereas Fig 1C and 1E show the average MS/MS scores ± S.E.M. obtained for each TRAPP subunit for *N* = 5 experiments)

MS/MS analysis of proteins pulled-down by Bet5-S (Fig 1E) showed that this bait co-purifies a mixture of TRAPPII, TRAPPIII and, potentially, TRAPPI. These proteins included the ‘core TRAPP hetero-heptamer’ subunits (Bet3A, Bet3B, Bet5, Trs23, Trs31, Trs20 and Trs33), the TRAPPII-specific subunits Trs120, Trs130 and Trs65, the TRAPPIII-specific subunit Trs85, and Tca17 (S1 Fig), which is an essential protein present in TRAPPII and TRAPPIII (see below). Notably, Bet5-S also pulled down the products of genes AN1374, AN4930 and AN4358 (http://www.aspgd.org), which similarity searches and domain analyses identified as *A*. *nidulans* homologues of mammalian TRAPPC11, TRAPPC12 and TRAPPC13, respectively (S1 Fig), establishing that the later three proteins are *bona fide* TRAPP components. Their physiological roles will be addressed elsewhere.

Purification with Trs120-S showed that in addition to the ‘core TRAPP hetero-heptamer’ and the TRAPPII-specific subunits Trs120, Trs130 and Trs65, TRAPPII contains Tca17 (Fig 1D and 1E), which was missing in the single particle EM yeast TRAPPII structure [30], but is a demonstrated yeast TRAPPII component [8,9]. The finding that TRAPPC11, TRAPPC12 and TRAPPC13 are absent from TRAPPII suggested that they belong to TRAPPIII, as in mammalian TRAPPs)[31–34], which is confirmed below. Thus, *A*. *nidulans* TRAPPs resemble those of metazoans to a greater extent than those of *S*. *cerevisiae*.

### Functional assignment of TRAPP subunits by lethality rescue analysis (LYRA)

TRAPP complexes mediate nucleotide exchange on RAB1 and RAB11, which play distinct and essential roles in the Golgi. Therefore genetic ablation of key TRAPP subunits Trs120, Trs130 and Bet3 results in lethality [7](S2 Fig). We analyzed all other TRAPP components (excepting TRAPPC11/12/13, which will be reported elsewhere). S2 Fig shows that single *bet5Δ*, *trs23Δ*, *trs31Δ*, *trs20Δ*, *tca17Δ* and double *trs33Δ trs65Δ* mutations also result in lethality, whereas *trs33Δ* and *trs65Δ* individually do not.

**Fig 2:**
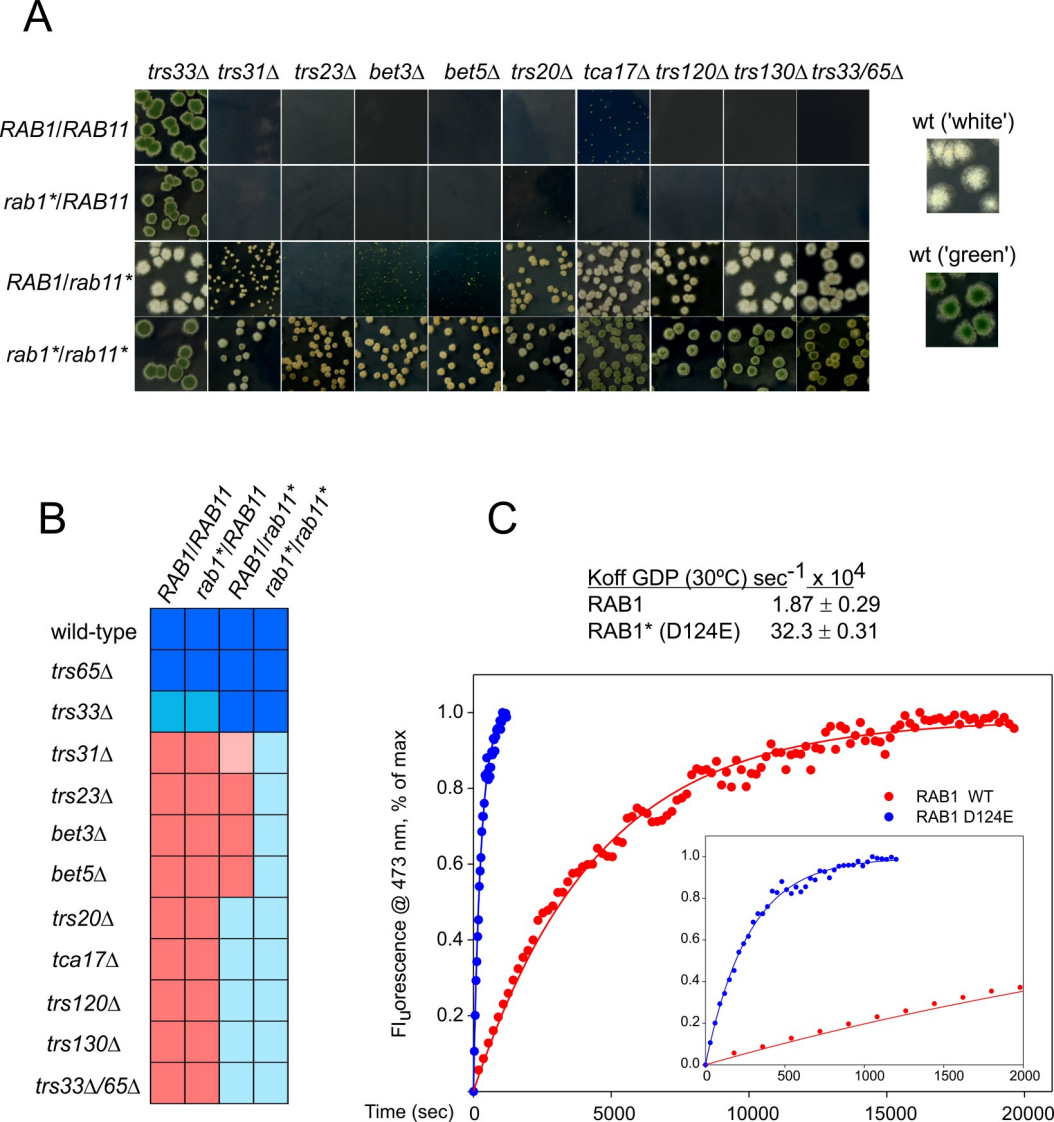
LYRA analysis of essential genes. (A) Heterokaryon rescue of lethal deletion alleles by constitutively active rab1* and rab11* alleles. Conidiospore suspensions were plated to give isolated colonies. For genotypes leading to lethal or severely debilitating phenotypes, conidiospores were obtained from heterokaryons. Wild-type colonies producing green or white conidiospores are shown on the right for comparison. (B) Color plot summarizing LYRA analysis. Dark blue represents wild-type growth whereas red indicates no growth; pink, very weak rescue; light blue, substantial rescue. (C) GDP release assays of wild-type and constitutively active D124E RAB1 (RAB1*). Time courses of mant-GDP exchange for GDP were used to calculate the observed *k*_*off*_ of GDP.

TRAPPII is the GEF for RAB11. *RAB11 (D125E)*, denoted rab11* for simplicity, encodes a mutant RAB11 having an Asp125Glu substitution that accelerates nucleotide exchange, thereby rescuing the lethality resulting from inactivating TRAPPII with *trs120Δ* or *trs130Δ* [7]. However, rab11* did not rescue *bet3Δ* [7] (Fig 2A and 2B). We hypothesized that rescuing the lethality resulting from *bet3Δ* would require combining rab11* with an equivalent *rab1* mutation. To this end we constructed rab1* encoding a mutant Asp124Glu RAB1. *In vitro* nucleotide exchange assays (Fig 2C) confirmed that Asp124Glu accelerates RAB1 GDP release by 17-fold, similar to equivalent Asp125Glu in RAB11 [7]. As expected, rab1* alone did not rescue *bet3Δ* lethality. However, *bet3Δ* cells were viable in the rab1* rab11* double mutant background, formally demonstrating that Bet3 (and thus TRAPPs) is (are) required for both RAB1 and RAB11 activation (Fig 2A and 2B). This result prompted us to use *l*ethalit*y r*escue *a*nalysis (LYRA) as a method to classify TRAPP components functionally: null mutants affecting essential subunits acting within TRAPPII should be rescued by rab11* alone, whereas those affecting subunits required for activating both RAB1 and RAB11 would necessitate rab1* plus rab11* to survive. Indeed *bet5Δ*, *trs23Δ* and *trs31Δ* removing any of the other subunits that are minimally required to mediate nucleotide exchange on both RAB1 and RAB11 [6] behaved like *bet3Δ* (*i*.*e*. necessitated both rab1* and rab11* to survive)(Fig 2A and 2B).

Trs20 is a component of TRAPPI, TRAPPII and TRAPPIII. In *S*. *cerevisiae* it has been shown that Trs20 links Trs120 to the TRAPP core complex, being required for the assembly of TRAPPII [35]. Although Trs20 is also present in TRAPPI/TRAPPIII, the viability of *A*. *nidulans trs20*Δ cells was rescued by rab11* alone, similarly to the prototypical TRAPPII mutants *trs120Δ* and *trs130Δ*, and the inclusion of rab1* did not improve *trs20*Δ growth any further. Therefore, these data show that Trs20 plays its essential physiological role with TRAPPII (Fig 2A and 2B), confirming the involvement of Trs20 in TRAPPII assembly deduced from the above *S*. *cerevisiae* studies [35](see below for studies of the instability of *A*. *nidulans trs20Δ* TRAPPII). They also agree with the reported interaction between human TRAPPC2 (Trs20) and TRAPPC9 (Trs120) [36]. As Trs20 is a TRAPPIII component [9](see below) the data also imply that Trs20 is not required by this complex for activating RAB1 in the secretory pathway.

It has been demonstrated that Trs20 also mediates the recruitment of Trs85 to TRAPPIII [36,37](see below for *A*. *nidulans*). As recently shown by Thomas et al. [9], TRAPPIII activates budding yeast RAB1 (Ypt1) in the secretory pathway. However Trs85 is not essential in *S*. *cerevisiae*, and, although the loss of Trs85 markedly diminishes RAB1 activation, it does not abolish it [9]. Thus, Trs85-less budding yeast TRAPPIII would suffice to provide sufficient levels of RAB1 activation during exocytosis to ensure viability [9]. On the other hand, yeast Trs85 mediates the RAB1-activating role of TRAPPIII during autophagy [38] [15,39], but this pathway does not operate under standard nutrient-sufficient conditions. In summary, the finding that *A*. *nidulans trs20Δ* is rescued by rab11* alone confirms that, as in budding yeast, Trs85 would not be essential for the secretory role of TRAPPIII, even though it would be required to target the complex for autophagy. (Loss of autophagy does not affect *A*. *nidulans* growth [40]).

Tca17, which is essential in *A*. *nidulans*, had not been functionally classified. Lethality resulting from *tca17*Δ was rescued by rab11* alone (Fig 2A and 2B), consistent with Tca17 being required for TRAPPII function, and arguing against its playing a role in RAB1 activation during exocytosis.

A similar conclusion was reached for Trs33 and Trs65. *S*. *cerevisiae trs33Δ* and *trs65Δ* are synthetically lethal, and the presence of at least one of them is required for the assembly of TRAPPII [41](see also below). *A*. *nidulans trs65Δ* does not affect growth, whereas *trs33Δ* causes a weak growth defect, suppressible by rab11* (Fig 2A and 2B and S2 Fig). As in yeast, *trs65*Δ and *trs33Δ* are synthetically lethal. rab11* rescued the viability of the double mutant as it did with *trs120Δ*, and the additional presence of rab1* did not improve growth further (Fig 2A and 2B). In summary, the essential roles of Tca17, Trs20, and of the Trs33/Trs65 pair reside in TRAPPII.

### How many TRAPP complexes are there in *A*. *nidulans*?

**Fig 3:**
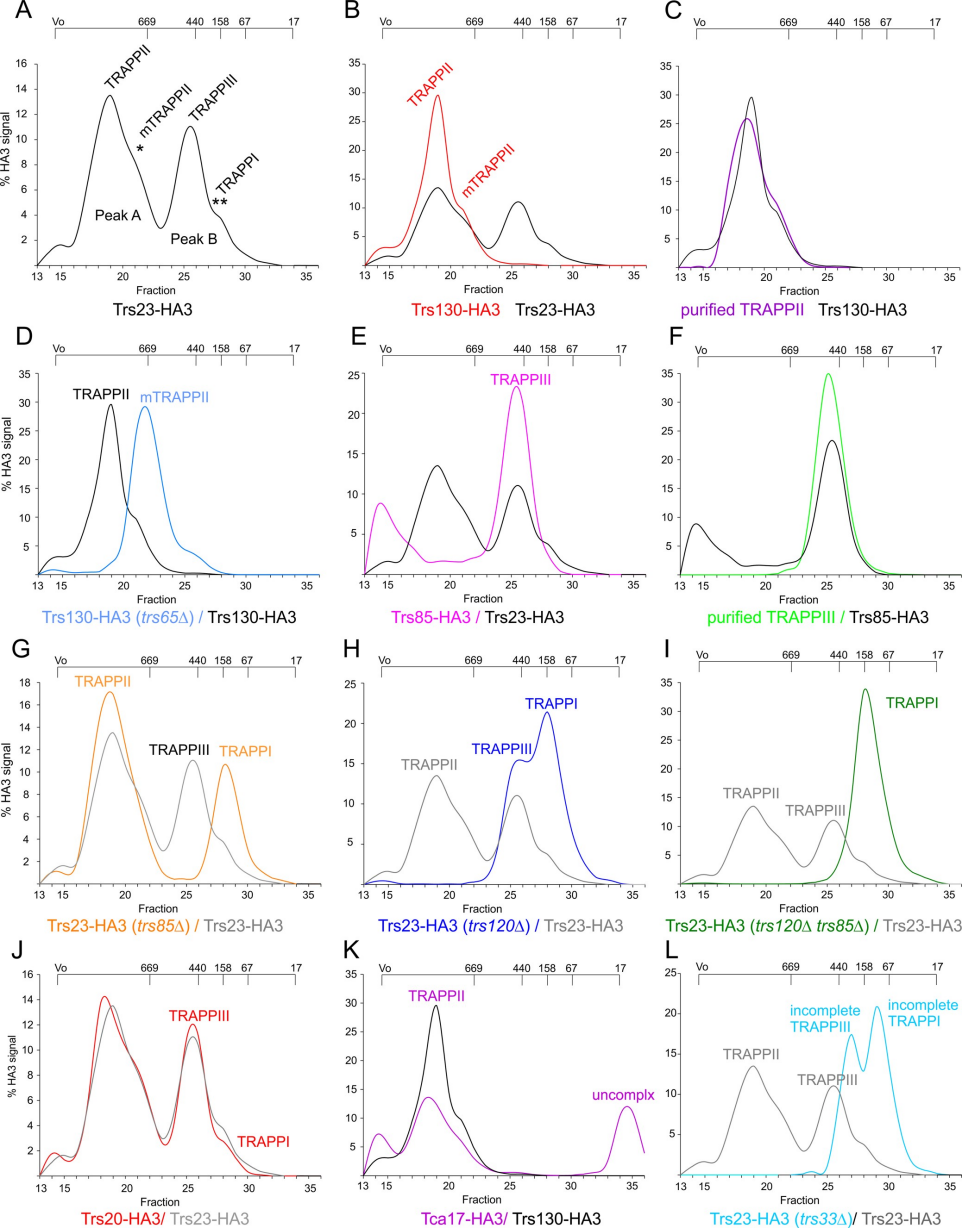
Gel filtration analysis of TRAPP complexes. Lysates prepared from the wt or the indicated mutants expressing endogenously HA-tagged TRAPP proteins were run through a Superose 6 column whose fractions were analyzed by anti-HA western blotting. Levels of each protein in any given fraction are plotted as percentage of the total signal in the column. The resulting elution profiles were smoothened using the ‘Simple Spline Curve’ option of SigmaPlot’s Graph menu. Elution positions of protein standards (in kDa) are indicated on the top. The identity of the different peaks is shown. (A) Wild-type Trs23-HA3 extracts. (B) Wild-type Trs130-HA3 extracts; reference (black), wild-type Trs23-HA3. (C) Purified TRAPPII (Trs23-HA3); reference (black), wild-type Trs130-HA3 extracts. (D) *trs65Δ* Trs130-HA3 extracts; reference (black) wild-type Trs130-HA3 extracts. (E) Wild-type Trs85-HA3 extract; reference (black), wild-type Trs23-HA3. (F) Purified TRAPPIII (Trs23-HA3); reference (black), Trs85-HA3. (G) *trs85Δ* Trs23-HA3 extracts; reference (black), Trs23-HA3 in the wild-type. (H) trs120Δ rab11* Trs23-HA3 extracts; reference (black), Trs23-HA3 in the wild-type. (I) trs85Δ trs120Δ rab11* Trs23-HA3 extracts; reference (black), Trs130-HA3 in the wild-type. (J) Wild-type Trs20-HA3 extracts; reference (grey), wild-type Trs23-HA3. (K) Wild-type Tca17-HA3 extracts; reference (black), wild-type Trs130-HA3 extracts. (L) *trs33Δ* Trs23-HA3 extracts; reference (black), wild-type Trs23-HA3 extracts.

MS/MS of Bet5-S-associated TRAPPs does not discriminate whether the core hetero-heptamer exists as an entity (TRAPPI) or solely as part of TRAPPII and TRAPPIII. To resolve TRAPP complexes present in cells we fractionated a crude lysate of an endogenously tagged Trs23-HA3 strain on a gel filtration chromatography column. Anti-HA western blotting of fractions revealed the existence of two major Trs23-containing peaks (Fig 3A). Peak A eluted at a position compatible with the ~ 1 MDa *Mr* corresponding to a TRAPPII homodimer — the main species of yeast TRAPPII purified from lysates [30] —(see also below). This peak had a shoulder of lower *Mr* representing the TRAPPII monomer (see below). Peak B eluted slightly ahead of the 440-kDa standard and also showed a shoulder (Fig 3A), whose elution position was consistent with that expected for TRAPPI, suggesting that TRAPPI might be present in this crude extract, although in a minor proportion (see below). An equivalent experiment using endogenously HA3-tagged core component Bet5 reproduced the pattern obtained with Trs23, validating these observations (S3 Fig).

**Fig 4:**
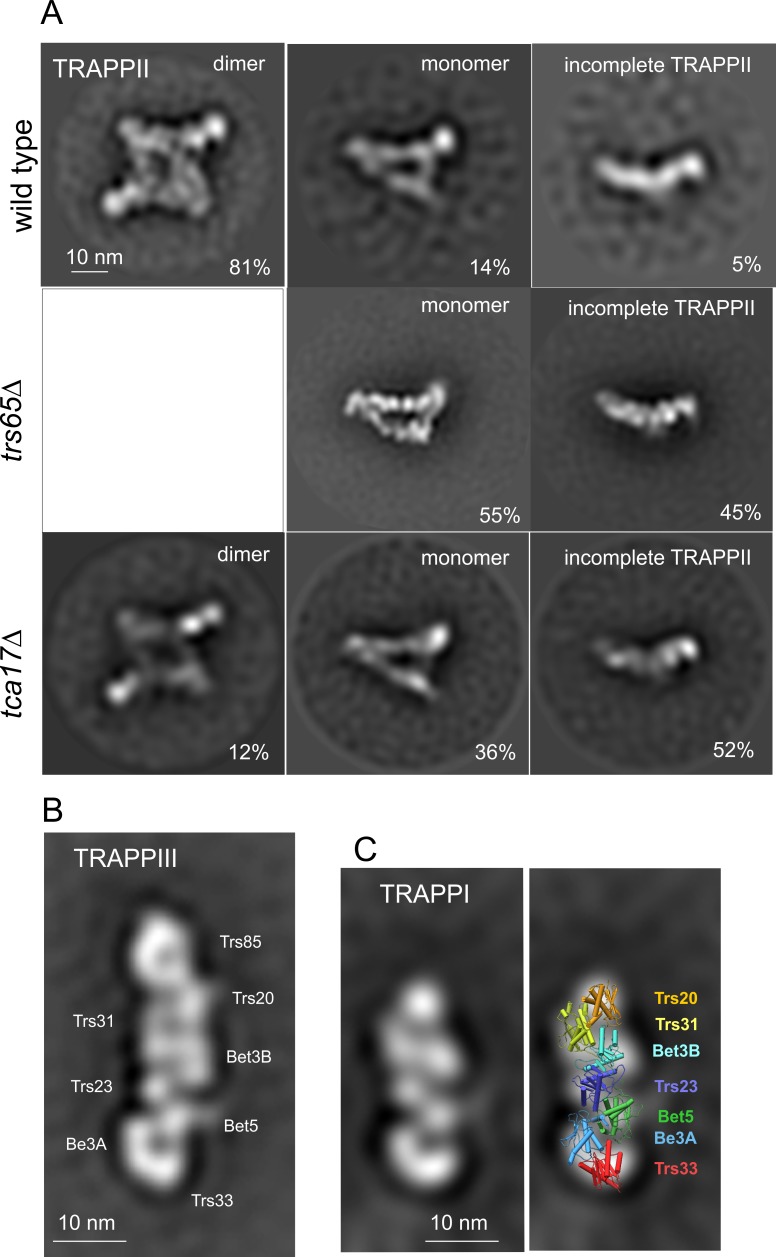
Negative staining 2D averages from purified TRAPP complexes. Complexes eluted from S-agarose columns were concentrated, stained with uranyl acetate and analyzed to obtain 2D class averages. (A) TRAPPII purified with Trs120-S from wild-type, *trs65Δ* and *tca17Δ* cells. The percentage of particles in each class is indicated. (B) TRAPPIII purified with Trs85-S from wild-type cells. The position of the components, based on previous studies, is indicated [37]. (C) Left, TRAPPI purified with Bet5-S from trs85Δ trs120Δ rab11* cells. Right, an atomic model generated from pdb crystal structures 3CUE, 2J3W and 2J3T [3,6] was manually superimposed onto the TRAPPI 2D class average.

Peak A represents TRAPPII, as established by the following facts: (i) Peak A was the only peak detected in crude extracts of an endogenously tagged Trs130-HA3 strain (Fig 3B); (ii) TRAPPII, purified by Trs120-S affinity chromatography as in Fig 1B, eluted at its position (Fig 3C); Peak A disappeared from crude extracts when Trs120 was ablated (Fig 3H, see below). Negative-staining EM followed by single particle 2D classification of Trs120-S-purified TRAPPII showed that it largely consists of a protein complex similar to the yeast TRAPPII dimer [30], with small proportions of triangle-shaped TRAPPII monomers and incomplete TRAPPII particles (Fig 4A, top)(see also below). Indeed the ‘shoulder’ of peak A represents the monomer, as demonstrated by the fact that *trs65Δ* preventing dimerization in yeast [30] shifted all of *A*. *nidulans* Trs130-HA3 TRAPPII present in crude extracts to the position of the ‘shoulder’ (Fig 3D) and markedly increased the proportion of monomers detected by EM in Trs120-S-purified TRAPPII preparations (Fig 4A, middle).

Peak B corresponds to TRAPPIII, as this was the only peak detectable in crude extracts of cells expressing Trs85-HA3 (Fig 3E). Moreover, TRAPPPIII, purified with S-tagged Trs85 as in Fig 1F and subsequently run through the gel filtration column eluted at the position of this peak (Fig 3F). MS/MS analysis of Trs85-S-purified TRAPPIII (*N* = 3 experiments) demonstrated that it consists of ‘core hetero-heptamer’ plus Trs85, Tca17 and TRAPPC11/TRAPPC12/TRAPPC13 (Fig 1F and 1G). However, their lower abundance strongly suggested that Tca17 and TRAPPC11/TRAPPC12/TRAPPC13 were being lost from the complex during purification for MS/MS. Indeed EM microscopy of Trs85-S TRAPPIII (requiring an additional concentration step) revealed a structure identical to that reported for yeast TRAPPIII, which lacks these four subunits (Fig 4B)[37].

The peak B shoulder detected in clarified lysates of strains expressing HA3-tagged core components elutes at the approximate position expected for core TRAPP hetero-heptamer (Trs33/Bet3A/Bet5/Trs23/Bet3B/Trs31/Trs20 account for ~155 kDa), suggesting that crude extracts might contain a minor amount of TRAPPI. To address this possibility we analyzed by gel filtration lysates of cells in which TRAPPII had been removed with *trs120Δ* (using rab11* to rescue lethality). Remarkably, the Trs23-HA3 TRAPPII peak in the wt was largely shifted to the position of the peak B shoulder in the mutant, with a lesser amount added to TRAPPIII (Fig 3H), which strongly indicates that this shoulder, present as a minor entity in crude wt extracts, corresponds to TRAPPI. In agreement, when we analyzed Trs23-HA in crude *trs85Δ* extracts TRAPPII was not altered, whereas TRAPPIII was shifted towards the position of the peak B shoulder (Fig 3G), further indicating that this shoulder represents TRAPPI. Definitive evidence supporting this conclusion came from analyzing TRAPPs present in clarified lysates of a double *trs120Δ trs85Δ* mutant lacking both TRAPPII and TRAPPIII (also using rab11* to rescue lethality), which showed that TRAPPs were completely shifted to the position of the peak B shoulder (Fig 3I). In a separate experiment we purified TRAPP from *trs120Δ trs85Δ* cells and analyzed the material by negative-staining EM. This analysis showed that Bet5-S-purified TRAPP from these double mutant cells corresponds to ‘core hetero-heptamer’ (i.e. TRAPPI)(Fig 4C). Thus, in *A*. *nidulans* extracts TRAPP exists in three versions: TRAPPII, the most abundant, TRAPPIII, compositionally similar to its metazoan counterpart and about half as abundant as TRAPPII and the low abundance core TRAPPI (i.e. the peak B shoulder).

Having determined the low abundance of TRAPPI in cell extracts we asked whether the two sedlin-like proteins Trs20 and Tca17 are stably associated to this core complex. Yeast Trs20 is present in TAP-purified *S*. *cerevisiae* TRAPPII and TRAPPIII [9,30], interacting with Trs120 and Trs85, respectively [35–37], as well as in S-tag-purified *A*. *nidulans* TRAPPII and TRAPPIII (Fig 1B–1G). When crude extracts of cells expressing Trs20-HA3 were analyzed by gel filtration, the elution profile observed for this protein was essentially identical to that of Trs23-HA3, specifically in the detection of the shoulder at the position of TRAPPI, indicating that Trs20 also associates with the core (Fig 3J) and agreeing with EM data (Fig 4C). In contrast Tca17-HA3 was only present in TRAPPII and in material eluting as uncomplexed protein (Fig 3K). Thus, all the above experiments support the contention that TRAPPI is a hetero-heptamer containing Trs20. On the other hand the substantial amount of uncomplexed Tca17 detected in extracts suggests that the association of Tca17 with TRAPPs is relatively weak. Experiments described below demonstrate that Tca17 is a component of TRAPPIII.

### Trs120 is crucial for TRAPPII assembly

**Fig 5:**
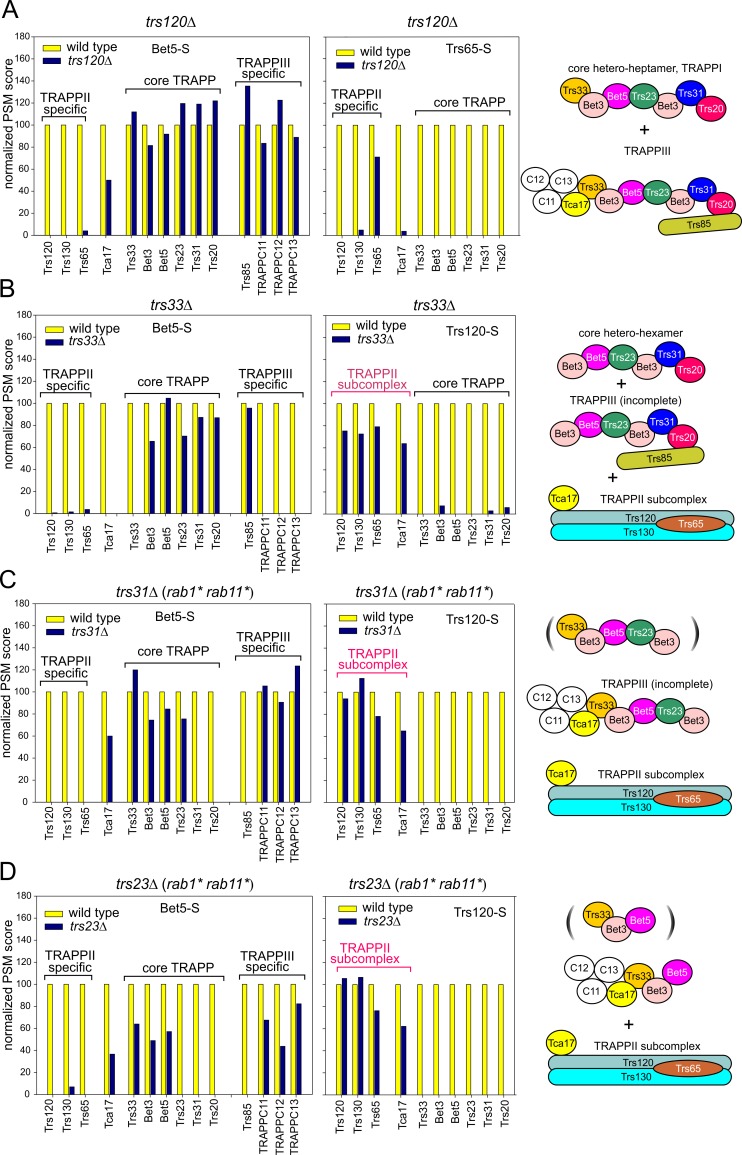
Composition of TRAPP complexes purified from *trs120Δ*, *trs33Δ*, *trs31Δ* and *trs23Δ* mutants analyzed by MS/MS. Parallel purifications of TRAPP complexes from wild-type and mutant cells were carried out using Trs120-S or Trs65-S (for TRAPPII) and Bet5-S (for all TRAPPs). Proteins eluted from the S-agarose resin with S-peptide were analyzed by shotgun MS/MS. For each mutant condition, the normalized protein scores obtained as in Fig 1 (i.e. spectral counts/number of Arg + Lys residues, multiplied by 100) were plotted relative to the corresponding scores in the wild-type, which were set as 100%. Diagrams on the right schematically depict the composition of TRAPP complexes. (A) Bet5-S and Trs65-S in *trs120*Δ cells. (B) Bet5-S and Trs120-S in *trs33Δ* cells. (C) Bet5-S and Trs120-S in *trs31Δ* cells rescued with rab1* and rab11*. (D) *trs23Δ* cells rescued with rab1* and rab11*. Whether the incomplete core complexes shown in brackets in the *trs31Δ* and *trs23Δ* mutants actually exist cannot be determined from available data.

Heretofore the effects of the absence of essential subunits on the architecture of TRAPP complexes could not be studied beyond bottom-up synthetic approaches with recombinant proteins. Therefore we exploited the ability to rescue genetically (with rab11* or with rab1* and rab11* combined) the viability of cells lacking essential genes to analyze native TRAPP complexes by MS/MS. To facilitate comparison, in parallel with the two experiments performed for each mutant condition (using *pan-*TRAPP Bet5-S and TRAPPII-specific Trs120-S or Trs65-S baits), we performed the corresponding experiments with the wt, and the data were plotted as percentage of PSM scores of each TRAPP component in the mutants compared to those in the wt. As proof of concept we investigated *trs120Δ* cells rescued with rab11*. MS/MS and gel filtration experiments (Figs 5A and 3H, respectively) showed that *trs120Δ* TRAPPs isolated with Bet5-S consisted of a mixture of TRAPPI and TRAPPIII. (Note that TRAPPIII-specific subunits co-purifying with Bet5-S include Trs85, TRAPPC11/TRAPPC12/TRAPPC13 and Tca17, reinforcing the conclusion that the latter is a TRAPPIII component) (Fig 1G). The absence of Trs130/Trs65 from the Bet5-S-purified material is consistent with the assembly of Trs130 being Trs120-dependent [5]. Indeed no other TRAPPII component was pulled-down using Trs65-S as TRAPPII-specific bait (Fig 5A, right), establishing that Trs120 is crucial for the assembly of TRAPPII and agreeing with our observation that the *trs120*^*ts*^ mutation *hypA1* destabilizes this complex [7].

### Trs33 is involved in the assembly of a TRAPPII-specific subcomplex onto core TRAPP

*A*. *nidulans trs33Δ* and *trs65Δ* are synthetically lethal, and LYRA assigned the essential overlapping role of Trs33 and Trs65 to TRAPPII (Fig 2A and 2B), in agreement with the report that yeast TRAPPII assembly requires Trs33 or Trs65 [41]. *trs65Δ* alone did not affect growth or the composition of the TRAPP complexes (S4 Fig), but prevented TRAPPII dimerization, as shown by gel filtration (Fig 3D) and EM (Fig 4A, middle) analyses, establishing that dimerization is not essential for TRAPPII function. *trs65Δ* also debilitated the assembly of TRAPPII-specific components with the core, as shown by the marked abundance of core-like particles detected by EM (Fig 4A, middle).

Single *trs33*Δ mutants showed a weak growth defect that was remediated by rab11* (S2 Fig), indicating that Trs33 acts, at least, with TRAPPII, agreeing with Trs33 contributing to the assembly of TRAPPII through direct interaction with Trs120 [41]. Bet5-S pulled-down from *trs33Δ* extracts a mixture of incomplete TRAPPI (lacking Trs33) and TRAPPIII (lacking Trs33, Tca17 and TRAPPC11/12/13) (Figs 3L and 5B). Thus Tca17 and TRAPPC11/12/13 recruitment to TRAPPIII requires Trs33 [Trs85 binds the core through Trs20, located opposite Trs33 [37]].

Notably, Bet5-S did not recover TRAPPII from *trs33Δ* extracts, but Trs120-S pulled-down a ‘subcomplex’ consisting of Trs120-S itself, Trs130, Trs65 and Tca17 (Fig 5B, right), indicating that Trs33 mediates the assembly of a ‘TRAPPII-specific subcomplex’ onto a TRAPPI core. The finding that *trs33Δ* debilitates the assembly of this Tca17-containing subcomplex onto the core confirms that Tca17 binds to the Trs33 end of the core hetero-heptamer, opposite to that of Trs20 (Fig 1A)[42–44]. Notably, although *trs33Δ* destabilizes TRAPPII *in vitro*, *trs33Δ* cells are viable, indicating that they contain some functional TRAPPII *in vivo*. In accordance, *trs33Δ* reduced the amount of TRAPPII present in TGN cisternae, monitored with Trs120-GFP, to 50% of the wt, but did not completely delocalize the complex (S5 Fig).

Interestingly, whereas the double *trs33Δ trs65Δ* mutation was indistinguishable from *trs33Δ* in the components copurifying with Bet5-S, no proteins copurified with Trs120-S in this background (S4 Fig), indicating that Trs65 is crucial for the assembly/stability of the TRAPPII-specific subcomplex. Thus, rather than Trs33 and Trs65 playing overlapping roles in TRAPPII assembly [41], the inefficient association of the TRAPPII-specific subcomplex with the TRAPP core (*trs33Δ*) combined with the inefficient assembly of the TRAPPII-specific subcomplex itself if Trs65 is additionally absent might underlie the lethality caused by *trs33Δ trs65*Δ.

### The TRAPPII-specific subcomplex coexists with incomplete core TRAPPs in cells lacking Trs31, Trs23 or Trs20

Next we investigated TRAPPs in cells lacking the core components Trs23 or Trs31, rescued with the rab1* rab11* mutant combination, aiming to disconnect ‘the Trs20 TRAPP side’ binding Trs85 and Trs120 [35,37] from the Bet5-S bait and Trs33.

Bet5-S pulled-down from *trs31Δ* extracts the three core components Bet3, Trs23 and Trs33, but not Trs20 (connected to Bet5 by Trs31 [3]), the TRAPPII-specific subunits Trs120/Trs130/Trs65 or the TRAPPIII-specific subunit Trs85 (Fig 5C, left). [Trs85 and Trs120 are recruited by Trs20 [35,37].] However, Bet5-S pulled Tca17 and TRAPPC11/TRAPPC12/TRAPPC13, consistent with their association with the core being Trs33-dependent. MS/MS cannot discriminate whether the pulled-down material consisted of incomplete TRAPPIII (lacking Trs85, Trs31 and Trs20) or a mixture of this with incomplete TRAPPI. However, this (these) complex(es) appear capable of activating RAB1 to some extent, as unlike *bet3Δ*, *bet5Δ* and *trs23Δ* mutations *trs31Δ* is weakly rescued by rab11* alone (Fig 2A and 2B). A similar TRAPP composition was obtained for *trs23Δ* (here only Bet3, Trs33 Tca17 and TRAPPC11/TRAPPC12/TRAPPC13 copurified with Bet5-S) (Fig 5D, right). Thus partial core hemi-complexes composed of Bet5, Bet3A and Trs33, with or without Trs23 and Bet3B, and decorated with Tca17 and TRAPPC11/TRAPPC12/TRAPPC13 are stable.

Notably, complementary Trs120-S experiments with *trs23Δ* or *trs31Δ* extracts pulled-down Trs120, Trs130, Trs65 and Tca17, i.e. the TRAPPII-specific subcomplex. That the pool of Tca17 is split between the incomplete *trs23Δ* or *trs31Δ* TRAPPIII complexes and the TRAPPII-specific subcomplex (Fig 5C and 5D) suggests that Tca17 acts at an intermediate stage in TRAPPII and TRAPPIII assembly. More importantly, these results indicated that the absence of Trs20, like that of Trs33 in the opposite side, prevents assembly of a stable TRAPPII-specific subcomplex onto the core TRAPP.

**Fig 6:**
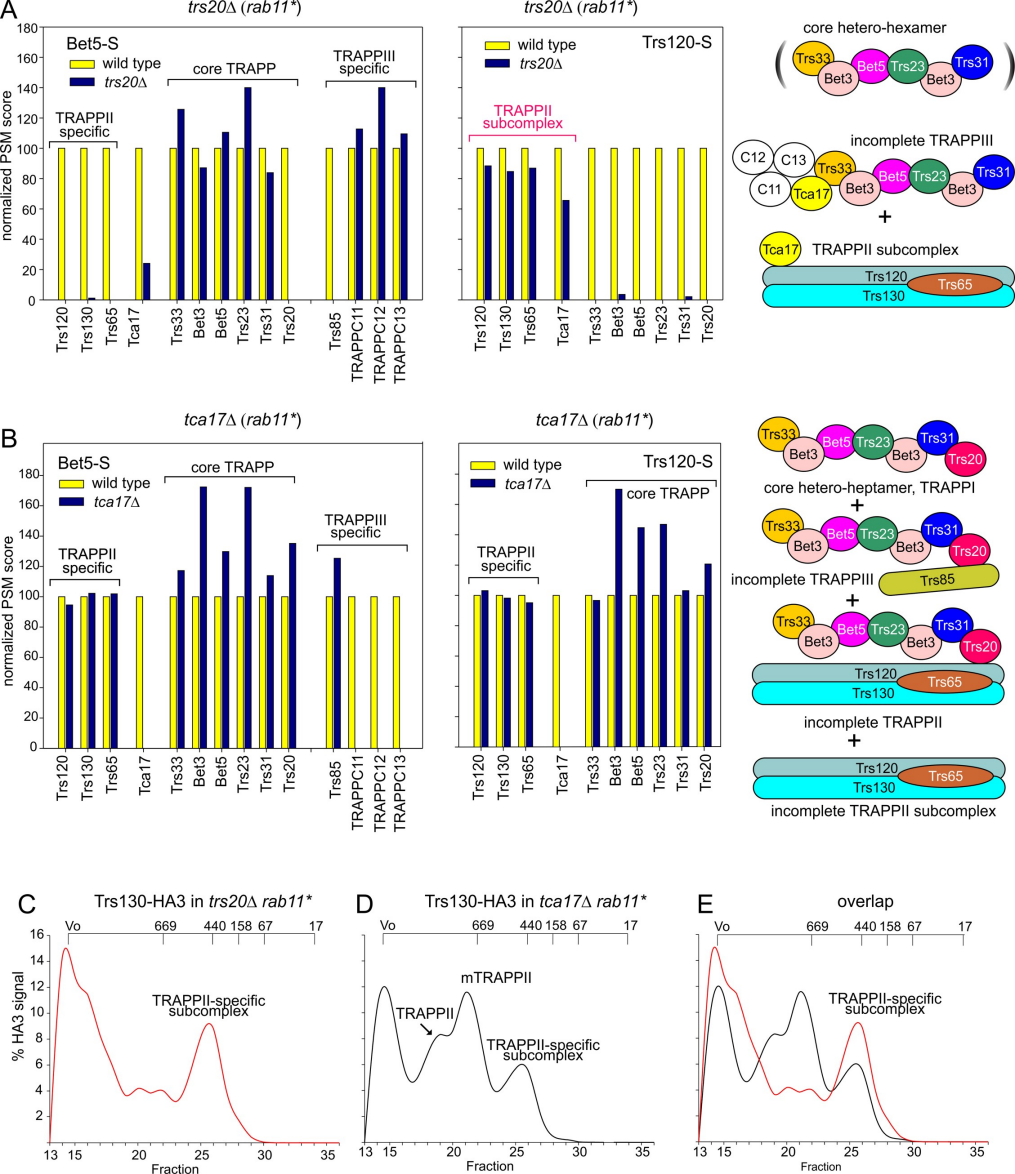
Composition of TRAPP complexes purified from *trs20Δ* and *tca17Δ* mutants: the TRAPPII-specific subcomplex. MS/MS analysis of purified TRAPP complexes as in Fig 5 and detection of the TRAPPII-specific subcomplex by gel filtration chromatography. (A) Shotgun proteomics of Bet5-S and Trs120-S complexes purified from *trs20Δ* cells rescued by rab11*. (B) As in (A) from *tca17Δ* cells also rescued by rab11*. (C) Gel filtration profile of trs20Δ rab11* Trs130-HA3 cell lysates. (D) Gel filtration profile of tca17Δ rab11* Trs130-HA3 cell lysates. (E) Gel filtration profiles of trs20Δ rab11* Trs130-HA3 and tca17Δ rab11* Trs130-HA3 cell lysates combined in the same plot for comparison. Elution profiles were smoothened using the ‘Simple Spline Curve’ option of SigmaPlot’s Graph menu, as in Fig 3. The identity of the different peaks is indicated. Note that under these mutant conditions a substantial amount of material, likely representing aggregates, elutes with the excluded volume.

To address this possibility we purified TRAPPs from rab11*-rescued *trs20*Δ cells. Bet5-S pulled the core complex, TRAPPC11/TRAPPC12/TRAPPC13 and some Tca17, but not Trs85 or Trs120/Trs130/Trs65 (Fig 6A). In sharp contrast, Trs120-S pulled down only Trs120/Trs130/Trs65 and Tca17, without core subunits (Fig 6A), establishing that the TRAPPII-specific subcomplex is a stable entity disconnected from the core by *trs20Δ*. Indeed Superose chromatography of crude lysates demonstrated that *trs20Δ* shifts Trs130-HA3 TRAPPII to a major peak of ~ 0.44 MDa, consistent with the *Mr* expected for the TRAPPII-specific subcomplex (Fig 6C). Thus, Trs20 facilitates the stable assembly of the TRAPPII-specific subcomplex onto the core, almost certainly by way of the reported interaction with Trs120 [35,36], playing a role similar to that of Trs33 acting at the opposite side of the core hetero-heptamer. The finding that Tca17 is stably attached to the TRAPPII-specific subcomplex detected in *trs20Δ*, *trs23Δ* and *trs31*Δ mutants (Figs 5A, 5C and 6A) agrees with reported interactions of Tca17 with Trs130 and Trs65 [13,45].

### The roles of Tca17 in TRAPPII and TRAPPIII

The presence of Tca17 in TRAPPs is Trs33-, but not Trs20-, Trs23-, Trs31- or Trs65-dependent (Figs 5 and 6 and S4 Fig), indicating that it requires Trs33 to bind the core, in agreement with reports suggesting that Tca17 binds to the opposite site of Trs20 in the core TRAPP hetero-heptamer [42–44].

Tca17 is a component of TRAPPII and TRAPPIII (Fig 1B and 1C). LYRA assigned the essential role of *A*. *nidulans* Tca17 to TRAPPII (Fig 2A and 2B). MS/MS of Trs120-S- purified TRAPP showed that *tca17Δ* (rescued with rab11*) does not affect TRAPPII composition beyond the absence of Tca17 itself (Fig 6B). However, in gel filtration experiments with crude extracts *tca17Δ* shifted the elution of Trs130/TRAPPII towards the positions of the monomer and of the TRAPPII-specific subcomplex, suggesting that it prevents TRAPPII dimerization and destabilizes the interaction of the latter with the core (Fig 6D and 6E). Indeed, single particle 2D classification of negatively-stained EM images of *tca17Δ* TRAPP purified with Trs120-S showed, in addition to a mixture of TRAPPII dimers (12%) and monomers (36%), an abnormally high proportion (52%) of incomplete TRAPPII particles (Fig 4A, bottom). This observation and the accumulation of the TRAPPII-specific subcomplex in this mutant (Fig 6D and 6E) strongly indicate that Tca17 ablation destabilizes the engagement of the TRAPPII-specific subcomplex with the core. *trs65Δ*, also preventing TRAPPII dimerization and markedly increasing the presence of incomplete TRAPPII particles (Fig 4A, middle), does not compromise TRAPPII stability to the same extent (compare Figs 3D and 6D) implying that Tca17 is more important than Trs65 to stabilize TRAPPII, in agreement with the fact that Tca17 is essential whereas Trs65 is not.

Tca17 is, in addition to a TRAPPII component, a TRAPPIII component (Fig 1F and 1G). Paradoxically, gel filtration analyses of crude lysates showed that Tca17 was exclusively complexed with TRAPPII (Fig 3K). However, the substantial proportion of uncomplexed Tca17 (Fig 3K) suggested that it is loosely associated to TRAPPIII, leading us to hypothesize that the salt concentration used (0.6 M) in these gel filtration experiments promoted its dissociation. Indeed, lowering salt concentration to 0.3 M revealed a minor Tca17 peak eluting in the TRAPPIII position (S6 Fig). Moreover, MS/MS analysis showed that Tca17-S co-purifies substantial amounts of Trs85, TRAPPC11, TRAPPC12 and TRAPPC13 from extracts (S7 Fig), confirming that a proportion of Tca17 is stably associated to TRAPPIII.

To investigate the role of Tca17 in TRAPPIII we analyzed by MS/MS the composition of Bet5-S-purified TRAPPs from *trs17Δ* extracts. In addition to TRAPPII-specific and core hetero-heptamer subunits, Bet5-S pulled Trs85, indicating that the absence of Tca17 does not completely destabilize TRAPPIII (Fig 6B). However, components TRAPPC11/TRAPPC12/TRAPPC13 were completely absent from this material (Fig 6B), implying that Tca17 is required for their recruitment to TRAPPIII, a conclusion fully consistent with their being also absent from Bet5-S-purified *trs33Δ* TRAPPs (Fig 5B.)(Recall that Trs33 recruits Tca17). That the amounts of Tca17 and TRAPPC11/12/13 present in Trs85-S-purified TRAPPIII appear substoichiometric (Fig 1F and 1G) suggests that these four subunits are loosely associated to TRAPPIII, in agreement with data above.

## Discussion

TRAPPs mediate nucleotide exchange on the essential RAB1 and RAB11 GTPases, and therefore most of their subunits are also essential. We exploited this for determining in which TRAPP complex(es) does each of these subunits play its role. Thus, rescuing lethality resulting from ablation of subunits playing an essential role with TRAPPII (Trs120, Trs130 Trs20, Tca17 and the Trs65/Trs33 combination) requires only constitutively active RAB11, implying that TRAPPII activates RAB11. In contrast, rescuing lethality resulting from ablating core TRAPP subunits Bet3, Bet5, Trs31 and Trs23 requires constitutively active both RAB1 and RAB11, implying that, in addition to being required by TRAPPII to activate RAB11, these core subunits are required by TRAPPI/TRAPPIII to activate RAB1 (but see further consideration of Trs31 below). Because ablation of all essential subunits could be suppressed by RAB mutations, it follows that the essential role of TRAPPs is mediating nucleotide exchange on RABs. However, in no case was growth of any lethal mutant restored to wild-type levels, suggesting that TRAPPs might play roles additional to mediating nucleotide exchange (activated RAB1 and RAB11 alleles do not themselves affect growth). While all these data would be compatible with RAB1 and RAB11 acting in two essential TRAPP-dependent parallel pathways, the interpretation consistent with current knowledge is that RAB1 and RAB11 act sequentially across the Golgi in the secretory pathway.

TRAPPII, regulating RAB11-mediated endocytic recycling maintaining hyphal tip growth [21], is the most abundant TRAPP. TRAPPII is dimeric, and dimerization requires Trs65 and, to a lesser extent, Tca17. However, unlike *trs65Δ*, *tca17Δ* results in lethality, which is rescued by constitutively active RAB11 alone, indicating that Tca17 plays an essential role in TRAPPII beyond dimerization. TRAPPII is composed of core hetero-heptamer plus Trs120/Trs130/Trs65 and Tca17, which assemble into a stable TRAPPII-specific subcomplex whose binding to the hetero-heptamer is destabilized by *trs20*Δ, *trs33*Δ or *tca17Δ*. We note that the existence of this stable Trs120/Trs130/Trs65/Tca17 TRAPPII-specific subcomplex might help resolving the as yet undetermined detailed molecular structure of TRAPPII.

TRAPPIII, half as abundant as TRAPPII, is composed of core TRAPP plus Trs85, Tca17 and TRAPPC11/TRAPPC12/TRAPPC13 binding TRAPPIII via Tca17. Thus Tca17 is a component of both TRAPPIII and TRAPPII, which is noteworthy because the metazoan Tca17 orthologue TRAPPC2L is also a TRAPPII/TRAPPIII component. Tca17 is not present in yeast TRAPPIII [9,15,37] and genes encoding the Tca17 interactors in TRAPPIII, TRAPPC11/TRAPPC12/TRAPPC13 are absent from the yeast genome, which has undergone a process of reductive evolution with extensive loss of genes and metabolic capabilities [26]. Thus, TRAPPIII composition indicates greater similarity between *A*. *nidulans* and metazoan TRAPPs. However, Tca17 and TRAPPC11/TRAPPC12/TRAPPC13 appear to bind loosely to TRAPPIII, as indicated by their substoichiometry in Trs85-S-purified TRAPPIII and their absence in EM samples requiring an additional step of purification. Alternatively, we cannot rule out the possibility that, *in vivo*, *A*. *nidulans* TRAPPIII be heterogeneous, with only a proportion having Tca17/TRAPPC11/12/13 associated with it. Others have proposed the existence of two different TRAPPIII complexes in yeast, according to whether they contain Trs33 or Trs85 [46]. However, in *A*. *nidulans* (Fig 1G) and yeast [9,37] Trs33 is a component of TRAPP complexes copurifying with Trs85.

TRAPPI (= core TRAPP) is a Tr33/Bet3A/Bet5/Trs23/Bet3B/Trs31/Trs20 hetero-heptamer. TRAPPI was thought to activate RAB1 on COPII vesicles/early Golgi cisternae [1,47]. However, TRAPPI does not exist in metazoans, and its presence as a functional entity in fungi has been questioned [9]. We detected TRAPPI as a minor peak by size-exclusion chromatography of wt crude extracts using Trs23, Bet5 or Trs20 immunodetection. Of note, mutations disorganizing TRAPPII, TRAPPIII or both commensurately shift Trs23 towards TRAPPI. TRAPPI contains Trs20, which according to LYRA is not required for the secretory role of RAB1. Thus, like Thomas et al. [9], we favor the possibility that this minor complex, although itself stable in the cell, results from dissociation of other TRAPPs either *in vivo* or during extract preparation. In any case, under-representation of TRAPPI gives further credence to the proposal that, as in mammalian cells, it is TRAPPIII that activates RAB1 during fungal exocytosis [9,48].

Lethality rescue by *rab* constitutive alleles enabled MS/MS analysis of mutant TRAPPs lacking essential components. Firstly *trs20Δ* cells lacking TRAPPII assemble the stable Trs120/Trs130/Trs65/Tca17 TRAPPII-specific subcomplex discussed above. Thus, in addition to recruiting Trs85 to TRAPPIII [37,39] (Fig 4B), Trs20, the human TRAPPC2 orthologue, crucially contributes to the assembly of the TRAPPII subcomplex onto the core, explaining its essential physiological role. Secondly, *trs23*Δ and *trs31*Δ mutants lacking key components of core TRAPP assemble the TRAPPII-specific subcomplex and ‘Bet5-containing subcomplexes’ previously undetected *in vivo* (Fig 5C and 5D). These TRAPPII-specific and Bet5-containing subcomplexes are disconnected from each other because the latter lack the major Trs120 interactor Trs20 [35]. Remarkably, the *trs31Δ* ‘Bet5-containing subcomplex‘ representing a much smaller yet stable ‘mini-core TRAPP’ appears capable of promoting some nucleotide exchange on RAB1, as the viability of *trs31Δ* cells can be weakly rescued by constitutively active RAB11 alone, contrasting with *trs23Δ*, *bet5Δ* or *bet3Δ*, which all require constitutively active RAB1 and RAB11 together.

Our data indicate that the two sedlin-like subunits, Tca17/TRAPPC2L and Trs20/TRAPPC2, facilitate the assembly of TRAPPII- and TRAPPIII-specific subunits onto the core, although Tca17 is not a stable component of the core. Studies with yeast Tca17 concluded that it cooperates with Trs33 and Trs65 to assemble/stabilize TRAPPII [42] and suggested the possibility that a TRAPPII subcomplex containing Trs120 and Trs130 is assembled onto core TRAPP through interaction with Trs20 on one side of the core and stabilization by Trs33 on the other [35]. We conclude that such stabilization of TRAPPII by Trs33 involves Tca17, a component of the TRAPPII-specific subcomplex that binds Trs33, located at the TRAPP end opposite to that of Trs20, which binds to the Trs120 component of the subcomplex. In contrast, in the pathway leading to TRAPPIII assembly Trs20 recruits Trs85 whereas Tca17 recruits TRAPPC11/TRAPPC12/TRAPPC13. This dual role of Tca17 in TRAPPII and TRAPPIII might help understand how homozygous mutations in the human homologue of Tca17, TRAPPC2L, result in a developmental disorder [45].

## Methods

### *Aspergillus* media, culture conditions and transgenes

Standard *A*. *nidulans* media were used for strain propagation and conidiospore production [49,50]. Genetic techniques have been detailed [51]. Transformation was as described [52]. Strains are listed in S1 Table.

### Endogenous tagging with S-tag an HA3 epitopes

Trs120-S has been described [7]. Genes encoding Bet5, Trs65, Trs85 and Tca17 were endogenously tagged with a C-terminal S-Tag by homologous recombination, using PCR-assembled cassettes [53,54] that contained, from 5’ to 3’, the C-terminal coding regions of the tagged genes (or the complete coding region, in the case of small genes) followed by the (Gly-Ala)5-S-Tag coding sequence (fused in frame to the tagged gene), the *Aspergillus fumigatus pyrG* gene and the corresponding 3’-flanking regions. *A*. *fumigatus pyrG* was used as selection marker for transformation of *nkuAΔ* strains deficient in the non-homologous end-joining recombination pathway [55]. Genes encoding Trs130, Tca17, Trs23, Bet5 and Trs85 were endogenously tagged with a C-terminal HA3 sequence (encoding a triplicated version of the HA epitope).

### S-agarose purification of TRAPPs

We modified a previous method [54,56]. Conidiospore suspensions of strains expressing Bet5-S, Trs120-S, Trs65-S, Trs85-S or Tca17-S from endogenously tagged alleles were inoculated in MFA (*Aspergillus* fermentation medium) consisting of SC supplemented with 2.5% (v/v) of corn steep liquor syrup (Solulys 048R, Roquette Laisa S.A., Valencia, Spain), 50 mM each of Na_2_HPO_4_ and NaH_2_PO_4_, 50 mM NaCl, 3% (w/v) sucrose as main C source, 20 mM (NH_4_)_2_SO_4_ as main N source and vitamins as required to supplement the auxotrophic mutations present in each strain. Cultures were incubated for 14–15 h at 30ºC with shaking and mycelia were collected by filtration (Miracloth, Millipore), washed with water and lyophilized in 50 ml Falcon tubes.

1.75 g of lyophilate were ground to a fine powder with 16 ceramic beads (MP Biomedicals, 1/4 inch diameter) and FastPrep homogenizer pulse of 20 sec at power 4. The powder was resuspended with 45 ml of extraction buffer [25 mM Tris-HCl, pH 7.5, 0.5% v/v NP40, 200 mM KCl, 4 mM EDTA, 1 mM DTT, 5 μM MG132, Complete ULTRA EDTA-free protease inhibitor cocktail (Roche)]. Then 5 ml of 0.6 mm glass beads were added and the mixture was homogenized with a 15 sec full-power pulse of the FastPrep followed by incubation for 10 min at 4ºC in a rotating wheel. This step was repeated two additional times before centrifuging the resulting homogenate at 15,000 x g for 30 min at 4ºC and collecting approximately 35 ml of supernatant, which was spiked with 1% (w/v) BSA. The solution was then combined with 0.5 ml of S-protein agarose (Novagen), equilibrated with extraction buffer containing 1% BSA, and the mixture was rotated for 2 h at 4ºC. Next agarose beads and bound proteins were collected by centrifugation for 2 min at 1400 rpm and 4ºC in an Eppendorf tabletop centrifuge. The resulting slurry was washed four times for 10 min at 4ºC with 10 ml of ice-cold ‘washing buffer’ containing 25 mM Tris pH 7.5, 300 mM KCl, 4 mM EDTA and 1 mM DTT, transferred to 2 ml Eppendorf tubes and resuspended in 0.5 ml of washing buffer containing 4 mg/ml of S-peptide. This tube was incubated for 15 min at 37ºC with shaking before collecting the supernatant after centrifugation in a microfuge. After repeating this step a second time, the two eluates were pooled, proteins were concentrated by 10% TCA (v/v) precipitation and microfuge centrifugation and the protein pellet was resuspended in 100 μl of Laemmli buffer. One third of this material was loaded onto a 10% SDS-polyacrylamide gel that was run until proteins moved ~ 1 cm into the separation gel. The portion of the gel containing the protein mixture (identified after staining with colloidal Coomassie) was excised and processed for MS/MS analysis.

### MS/MS analysis

Tryptic peptide identification was carried out using an Easy-nLC 1000 nano-flow chromatograph (Thermo Fisher) coupled to a Q Exactive mass spectrophotometer. MS data were analyzed with Proteome Discoverer (version 1.4.0.288) (Thermo). Mass spectra *.raw files were searched against *Aspergillus nidulans* FGSC A4 version_s10m02-r03_orf_trans_allMODI (8223 protein sequence entries) using the Sequest search engine. Identified peptides were filtered using Percolator [57] with a q-value threshold of 0.01. For Fig 1 and S7 Fig, the abundance of each protein hit was given as the number of PSMs for each TRAPP protein divided by the number of Lys + Arg residues present in the protein sequence and multiplied by 100. For all other Figs involving parallel purifications from wt and mutant cells these scores were plotted as % relative to the wt.

### Superose 6 size exclusion chromatography

80 mg of lyophilized mycelia, obtained from MFA cultures as above, were ground in 2 ml screw-cap microcentrifuge tubes containing a ceramic bead with a 20 sec pulse of FastPrep set at power 4. The resulting powder was resuspended in 1.5 ml of lysis buffer containing 25 mM Tris-HCl, pH 7.5, 0.5% v/v NP40, 600 mM KCl, 4 mM EDTA, 1 mM DTT, 5 μM MG132 and Complete ULTRA EDTA-free protease inhibitor cocktail (Roche). ~ 100 μl of 0.6 mm glass beads were added to the suspension, which was homogenized with a 15 sec pulse at full power of the FastPrep followed by incubation for 10 min at 4ºC in a rotating wheel. This step was repeated two additional times before the resulting homogenate was centrifuged at 15,000 x g and 4ºC in a refrigerated microcentrifuge. 200 μl of the supernatant were loaded onto a Superose 6 10/300 column (GE Healthcare) equilibrated in 0.6 M KCl lysis buffer, which was run at a flow rate of 0.5 ml/min in an ÄKTA FPLC (Amersham-Pharmacia Biotech). Fractions of 0.5 ml were collected. The column was calibrated with the following standards: Myoglobin (17 kDa), BSA (67 kDa), aldolase (158 kDa) ferritin (449 kDa) and thyroglobulin (669 kDa). The exclusion volume (Vo) was determined with dextran blue.

Twenty-three fractions of 0.5 μl starting from the excluded volume were analyzed: 80 μl of each were mixed with 40 μl of 2-times concentrated Laemmli loading buffer, denatured at 100ºC and 25 μl of the mixture were loaded onto 10% SDS-PAGE gel, which was transferred to nitrocellulose and analyzed by western blotting using anti-HA Roche rat monoclonal 3F10 (1/1000) and HRP-coupled goat anti-rat (Southern Biotech, 1/4000) as primary and secondary antibodies, respectively. HRP was detected with Clarity western ECL substrate (Biorad). Chemiluminescence was recorded with a Biorad Chemidoc and the signal in anti-HA-reacting bands was quantified with Image Lab 5.2.1 (Biorad) and plotted with SigmaPlot. Elution profiles were smoothened using the ‘Simple Spline Curve’ option of the Graph menu.

### GDP exchange assays

Wild-type and D124E GST-RAB1 proteins were expressed in *E*. *coli*, purified on glutathione-Sepharose 4B and loaded with GDP as described [7]. 5 μM GDP-loaded fusion proteins [7] were incubated at 30ºC in a buffer containing 50 mM Tris-HCl, pH 7.5, 10 mM MgCl_2_, 10 mM KCl, 2 mM DTT and 50 μM mant-GDP. The exchange of GDP by mant-GDP was monitored in a fluorimeter (Jobin-Ybon SPEX Fluoromax-2; HORIBA, Ltd.) as the increase in fluorescence emission at 437 nm resulting from exciting Trp residues in the RABs at 290 nm and subsequent FRET transfer to the methylanthraniloyl group of mant-GDP present in the nucleotide-binding pocket. [58]. The excess of mant-GTP in the reactions results in pseudo-first order conditions in which the increase of fluorescence can be fitted to an exponential curve from which the *Koff* could be derived [7] (SigmaPlot 11.0, Systat Software). The values presented in Fig 2C are the average of 4 different experiments.

### Gene deletions, heterokaryon rescue and LYRA

Single gene deletion alleles were constructed by transformation of *nkuAΔ pyrG89* MAD5736 or MAD6512 recipient strains (S1 Table) with DNA cassettes assembled by PCR [53]. These consisted of approximately 800–900 bp fragments upstream of the coding ATG and downstream of the translation termination codon, flanking the *A*. *fumigatus pyrG* gene used as selective marker, such that the complete coding region of the gene was deleted after homology-mediated double cross-over [55]. In the case of *trs33Δ*, we also used a cassette based on *A*. *fumigatus riboB* gene, which was transformed into a *riboB2 pyrG89* strain (MAD5319). The resulting *trs33Δ*::*riboB pyrG89* strain was used as recipient of a second transformation with the *trs65Δ*::*pyrG* cassette. Nuclei carrying single or double deletion alleles resulting in lethality were recovered in heterokaryosis [59] (see S2 Fig). The presence of the deletions in heterokaryons was in all cases confirmed by diagnostic PCR using primers flanking the deletion cassette [7].

The rab1* allele was constructed after homologous recombination-mediated replacement of the endogenous *rab1* gene in MAD5319 (S1 Table) by a DNA cassette consisting of the *rab1* 5’-flanking region (200 bp), the (mutant) complete *rab1* coding region (cDNA version), 150 pb of the *rab1* 3’-flanking region, the *A*. *fumigatus riboB* gene and a further 800 bp of *rab1* 3’-flanking region [24]. The mutant *rab1* coding region in this cassette contained a T372G transversion resulting in Asp124Glu substitution. *pyrG* deletion cassettes were introduced into the rab1*::*riboB*^*+*^ *pyrG89* background by transformation of MAD6101 (S1 Table). rab11* is the classical *rabE28* allele resulting in Asp125Glu substitution [7]. *pyrG* deletion cassettes were introduced into the rab11* pyrG89 background by transformation of MAD5728 (S1 Table). A rab1* rab11* pyrG89 strain (MAD6160) suitable for introducing *pyrG* deletion cassettes was obtained from the progeny of a cross between strains MAD4919 and MAD6101. The presence of rab11* was diagnosed by DNA sequencing, whereas that of rab1* was determined by diagnostic PCR with flanking primers.

Heterokaryotic primary transformants were subcultured at 37ºC onto pyrimidine-less MCA plates. Conidiospores harvested from these heterokaryons were used for diagnostic PCR of deletion alleles; the effects of null alleles on viability were tested by plating equivalent amounts of these conidiospores on SC plates containing or not pyrimidines. Conidiospores gave rise to colonies on pyrimidine-less SC medium only if the *pyrG*^*+*^ transformed nuclei were viable, which for TRAPP lethal mutations occurred only if the single or double *rab* mutants were able to rescue viability (S2A Fig).

### Negative-stain electron microscopy and image processing

S-agarose purified TRAPPs were eluted with 0.5 ml of elution buffer containing S-peptide as above. These samples were concentrated to 100 μl using Amicon Ultra centrifugal filters (0.5 ml, 30 kDa cut-off; 6500 rpm and 4ºC for 15–20 min in a microcentrifuge). Immediately after purification, 4 μl of fresh complexes were applied to glow-discharged continuous carbon grids. After 1 min incubation the grids were quickly washed with two consecutive 40 μl drops of milli-Q water, and stained with two 10 μl drops of 2% uranyl acetate during 1 min prior blotting completely dry. Negatively-stained specimens were examined using a JEOL-1230 electron microscope operated at 100 kV acceleration voltage and equipped with a Tem-Cam-F416 4k x 4k pixel camera (TVIPS, Gauting, Germany). Data were acquired using a dose of ~25 e^-^/Å^2^ at a nominal magnification of 40,000 x, which correspond to 2.8 Å/px at the specimen level. For image processing, individual particles (6,717 wt TRAPPII; 22,017 *trs65*Δ TRAPPII; 32,245 *tca17*Δ TAPPII; 35,621 Trs85-S TRAPPIII; 21,292 Bet5-S *trs120Δ trs85Δ* TRAPPI) were semi-automatically picked, extracted and phase-flipped with EMAN2 [60], and subjected to 2D classification using RELION [61].

### Fluorescence microscopy

Wide-field fluorescence microscopy was carried out as previously detailed [17]. To determine the effects of *trs33Δ* in the recruitment of Trs120-GFP to TGN cisternae, 5 μm-deep Z-stacks of wt and *trs33Δ* cells expressing Trs120-GFP were acquired with the same settings. Maximal intensity projections of these images were thresholded to include only the fluorescence signal present in Golgi puncta in the apicalmost 20 μm of the hyphae and the total amount of signal within the thresholded area was calculated and plotted with Prism 3.02.

## Supporting information

S1 Fig Identification an structure of TRAPP genes previously missing in the database. (A) Gene structure and translation products of Tca17 and Trs23. Coordinates and systematic ASPN_ gene calls were obtained from the *A*. *nidulans* genomic sequence of the Centre for Structural and Functional Biology at Concordia University in Montreal, CA. (http://www.fungalgenomics.ca). Tca17 is a 175-residue (19 kDa) protein containing a Sedlin_N PF04628 domain. Tca17 is essential for growth (S2 Fig). Trs23 is a 199-residue (21.8 kDa) protein containing two regions that match Sybindin PF04099 domains (related to Sedlin_N domains). (B) BLAST searches of the *A*. *nidulans* AspGD genomic database (http://www.aspgd.org/) identified genes encoding homologues of metazoan TRAPPC11 (AN1374; 1258-residue and 141.8 kDa; contains Foie-gras and Gryzun domains), TRAPPC12 (AN4930; 445-residue and 49.2 kDa; contains C-terminal TPR domain and matches PTHR21581: SF6 ‘trafficking protein particle complex subunit 12’) and TRAPPC13 (AN4358; 334-residue, 36.7 kDa; contains a PFAM06159 domain and matches the Interpro IPR010378 ‘trafficking protein particle complex subunit 13’ family. The intron structure of these genes is correctly annotated in AspGD.
(PDF)

S2 Fig Essential and non-essential proteins in TRAPPs by deletion analysis and heterokaryon rescue. (A) Top left scheme. Nuclei carrying a deletion allele of an essential gene that has been replaced by an *Aspergillus fumigatus pyrG*^*+*^ allele (red, *delta*::*pyrG*^*+*^) after transformation and homologous recombination can be maintained in heterokaryosis with wild-type nuclei of the recipient strain carrying a pyrimidine-requiring *pyrG*^*-*^ mutation (green, *pyrG*^*-*^). As individual nuclei segregate into conidiospores, no conidiospores isolated from colonies of this heterokaryotic strain will grow on media lacking pyrimidines if the deleted gene is essential (the only prototrophic conidiospores will carry the lethal deletion allele), although, as a control, the wild-type *pyrG*^*-*^ conidiospores will grow on medium supplemented with pyrimidines. The Petri dishes show this test performed on the indicated gene deletion strains, whose genotype had been confirmed by diagnostic PCR of the heterokaryons. Colonies obtained from wild-type strains carrying or not the *wA2* mutation resulting in white conidiospores are shown as reference. (B) Colonies of wild-type controls and of *trs33Δ* strains carrying, where indicated, rab1*, rab11* or both.
(PDF)

S3 Fig Gel filtration analysis of Bet5-HA3 TRAPPs. A lysate prepared from a wild-type strain expressing endogenously tagged Bet5-HA3 was run through a Superose 6 column and the elution profile was subsequently monitored by anti-HA western blotting of fractions. Levels of the protein in any given fraction are represented as percentage of the total signal in the column. The elution profile was smoothened using the ‘Simple Spline Curve’ option of SigmaPlot’s Graph menu. Elution positions of protein standards (in kDa) are indicated on the top.
(PDF)

S4 Fig MS/MS shotgun analyses of trs33Δ, trs65Δ and the double mutant strains. Parallel S-agarose affinity purifications of TRAPP complexes from wild-type and indicated mutant cells were carried out using Trs120-S (TRAPPII) and Bet5-S (all TRAPPs). Proteins eluted from the S-agarose resin were analyzed by shotgun MS/MS. For each mutant condition, the PSM scores obtained as in Fig 5 were plotted as bar diagrams relative to the corresponding scores in the wild-type, which were set as 100%. Diagrams on the right represent the schematic composition of TRAPP complexes. (A) Bet5-S and Trs120-S in *trs33*Δ cells. (Note that this plots are the same as those displayed in Fig 5B, and are additionally shown here for convenience, to facilitate comparison with those shown in panels B and C.) (B) Bet5-S and Trs120-S in *trs65Δ* cells. (C) Bet5-S and Trs120-S in *trs33Δ trs65Δ* cells rescued with rab1* and rab11*.
(PDF)

S5 Fig *trs33*Δ partially delocalizes Trs120. Wild-type and *trs33Δ* cells expressing Trs120-GFP. The images (shown in both colour and inverted greyscale for clarity) are maximal intensity projections. The graph on the right shows the quantitation of the Trs120-GFP fluorescence signal in TGN cisternae of *N =* 13 wild-type and *N =* 12 in *trs33Δ* cells, respectively. The two datasets differ significantly as determined by an unpaired *t*-test.
(PDF)

S6 Fig Tca17-containing TRAPPs at different KCl concentrations. The elution profiles on Superose 6 of TRAPPs monitored with Tca17-HA3 are shown. The experiments were carried out using 300 mM or 600 mM KCl to illustrate the fact that TRAPPIII containing Tca17 becomes detectable if the salt concentration is reduced, indicating that Tca17 is loosely associated to the complex. Elution profiles were smoothened using the ‘Simple Spline Curve’ option of SigmaPlot’s Graph menu.
(PDF)

S7 Fig MS/MS shotgun of Tca17-containing TRAPP complexes. The composition of TRAPP complexes copurifying with Tca17-S in an affinity column was determined by shotgun sequencing. Note that all TRAPPIII-specific proteins including Trs85 copurify with Tca17. The scheme is an interpretation of the complexes containing Tca17.
(PDF)

S1 Table Strains used in this work (2 pages). (PDF)
