# Supplementary material for: Characterization of Aspergillus nidulans TRAPPs uncovers unprecedented similarities between fungi and metazoans and reveals the modular assembly of TRAPPII
Source: PLoS Genet. 2019 Dec 23;15(12):e1008557. doi: 10.1371/journal.pgen.1008557 (PMC6946167; doi:10.1371/journal.pgen.1008557)
Supplement: S1 Fig — (A) Gene structure and translation products of Tca17 and Trs23. Coordinates and systematic ASPN_ gene calls were obtained from the A. nidulans genomic sequence of the Centre for Structural and Functional Biology at Concordia University in Montreal, CA. (http://www.fungalgenomics.ca). Tca17 is a 175-residue (19 kDa) protein containing a Sedlin_N PF04628 domain. Tca17 is essential for growth (S2 Fig). Trs23 is a 199-residue (21.8 kDa) protein containing two regions that match Sybindin PF04099 domains (related to Sedlin_N domains). (B) BLAST searches of the A. nidulans AspGD genomic database (http://www.aspgd.org/) identified genes encoding homologues of metazoan TRAPPC11 (AN1374; 1258-residue and 141.8 kDa; contains Foie-gras and Gryzun domains), TRAPPC12 (AN4930; 445-residue and 49.2 kDa; contains C-terminal TPR domain and matches PTHR21581: SF6 ‘trafficking protein particle complex subunit 12’) and TRAPPC13 (AN4358; 334-residue, 36.7 kDa; contains a PFAM06159 domain and matches the Interpro IPR010378 ‘trafficking protein particle complex subunit 13’ family. The intron structure of these genes is correctly annotated in AspGD. (PDF) [file pgen.1008557.s001.pdf]

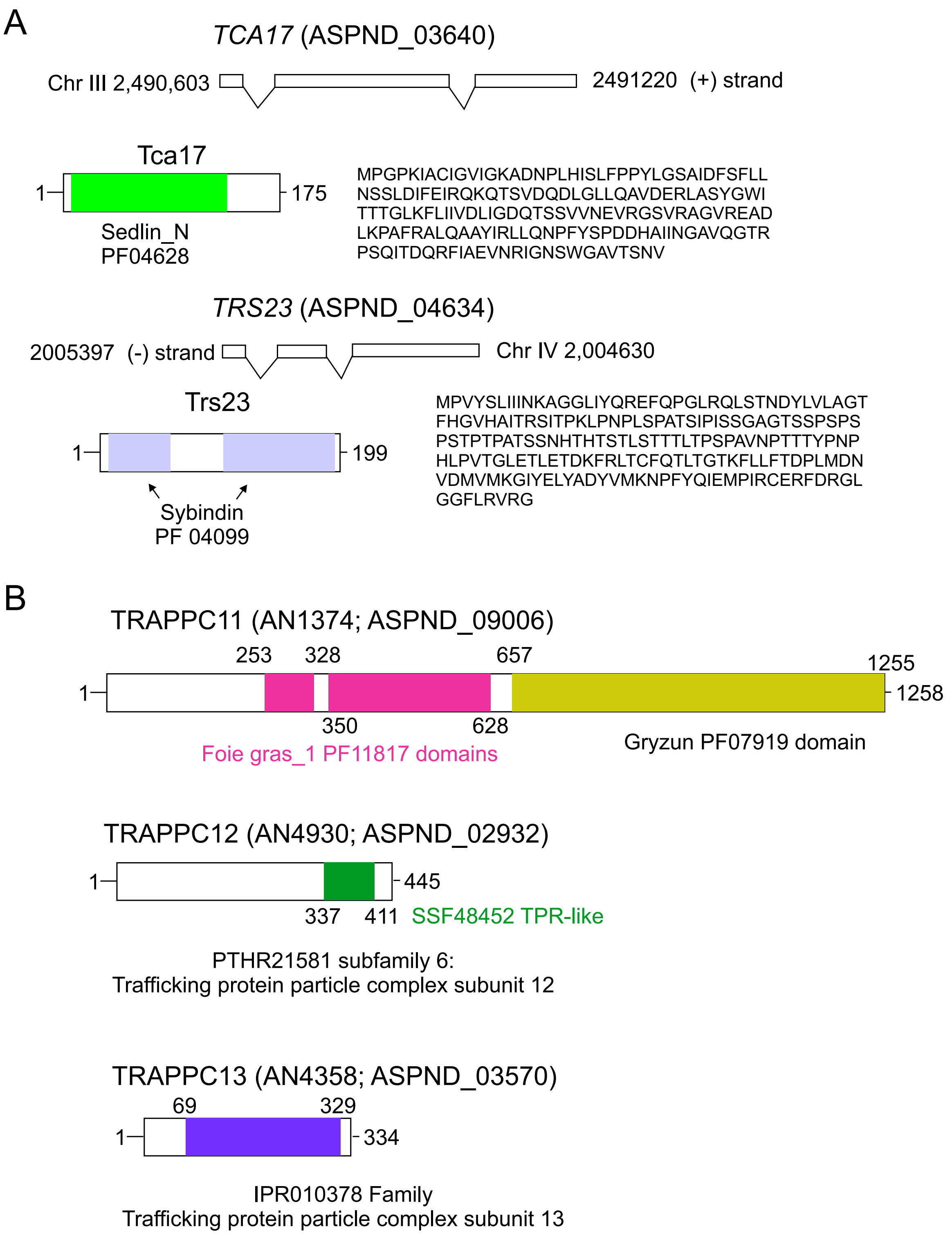

S1 Fig. Identification an structure of TRAPP genes previously missing in the database
(A) Gene structure and translation products of Tca17 and Trs23. Coordinates and systematic ASPN_ gene calls were obtained from the *A. nidulans* genomic sequence of the Centre for Structural and Functional Biology at Concordia University in Montreal, CA. (http://www.fungalgenomics.ca). Tca17 is a 175-residue (19 kDa) protein containing a Sedlin_N PF04628 domain. Tca17 is essential for growth Trs23 is a 199-residue (21.8 kDa) protein containing two regions that match Sybindin PF04099 domains (related to Sedlin_N domains). (B) BLAST searches of the *A. nidulans* AspGD genomic database (http://www.aspgd.org/) identified genes encoding homologues of metazoan TRAPPC11 (AN1374; 1258-residue and 141.8 kDa; contains Foie-gras and Gryzun domains), TRAPPC12 (AN4930; 445-residue and 49.2 kDa; contains C-terminal TPR domain and matches PTHR21581: SF6 'trafficking protein particle complex subunit 12') and TRAPPC13 (AN4358; 334-residue, 36.7 kDa; contains a PFAM06159 domain and matches the Interpro IPR010378 'trafficking protein particle complex subunit 13' family. The intron structure of these genes is correctly annotated in AspGD.
