# Supplementary material for: Characterization of Aspergillus nidulans TRAPPs uncovers unprecedented similarities between fungi and metazoans and reveals the modular assembly of TRAPPII
Source: PLoS Genet. 2019 Dec 23;15(12):e1008557. doi: 10.1371/journal.pgen.1008557 (PMC6946167; doi:10.1371/journal.pgen.1008557)
Supplement: S2 Fig — (A) Top left scheme. Nuclei carrying a deletion allele of an essential gene that has been replaced by an Aspergillus fumigatus pyrG+ allele (red, delta::pyrG+) after transformation and homologous recombination can be maintained in heterokaryosis with wild-type nuclei of the recipient strain carrying a pyrimidine-requiring pyrG- mutation (green, pyrG-). As individual nuclei segregate into conidiospores, no conidiospores isolated from colonies of this heterokaryotic strain will grow on media lacking pyrimidines if the deleted gene is essential (the only prototrophic conidiospores will carry the lethal deletion allele), although, as a control, the wild-type pyrG- conidiospores will grow on medium supplemented with pyrimidines. The Petri dishes show this test performed on the indicated gene deletion strains, whose genotype had been confirmed by diagnostic PCR of the heterokaryons. Colonies obtained from wild-type strains carrying or not the wA2 mutation resulting in white conidiospores are shown as reference. (B) Colonies of wild-type controls and of trs33Δ strains carrying, where indicated, rab1*, rab11* or both. (PDF) [file pgen.1008557.s002.pdf]

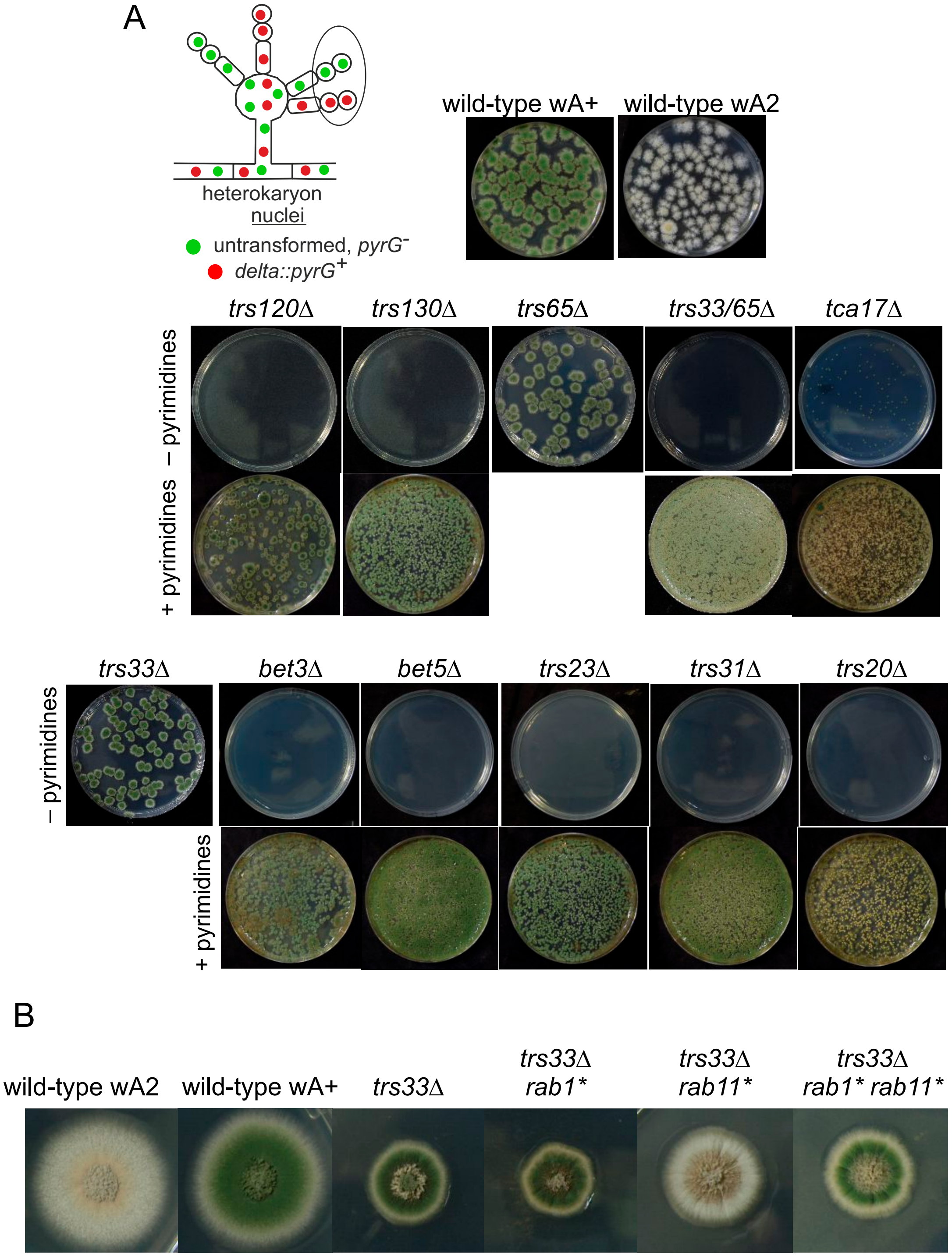

S2 Fig. Essential and non-essential proteins in TRAPPs by deletion analysis and heterokaryon rescue. (A) Top left scheme. Nuclei carrying a deletion allele of an essential gene that has been replaced by an *Aspergillus fumigatus pyrG*$^{+}$ allele (red, *delta::pyrG*$^{+}$) after transformation and homologous recombination can be maintained in heterokaryosis with wild-type nuclei of the recipient strain carrying a pyrimidine-requiring *pyrG*$^{-}$ mutation (green, *pyrG*$^{-}$). As individual nuclei segregate into conidiospores, no conidiospores isolated from colonies of this heterokaryotic strain will grow on media lacking pyrimidines if the deleted gene is essential (the only prototrophic conidiospores will carry the lethal deletion allele), although, as a control, the wild-type *pyrG*$^{-}$ conidiospores will grow on medium supplemented with pyrimidines. The Petri dishes show this test performed on the indicated gene deletion strains, whose genotype had been confirmed by diagnostic PCR of the heterokaryons. Colonies obtained from wild-type strains carrying or not the *wA2* mutation resulting in white conidiospores are shown as reference. (B) Colonies of wild-type controls and of *trs33*Δ strains carrying, where indicated, *rab1**, *rab11** or both.
