# Supplementary material for: Characterization of Aspergillus nidulans TRAPPs uncovers unprecedented similarities between fungi and metazoans and reveals the modular assembly of TRAPPII
Source: PLoS Genet. 2019 Dec 23;15(12):e1008557. doi: 10.1371/journal.pgen.1008557 (PMC6946167; doi:10.1371/journal.pgen.1008557)
Supplement: S3 Fig — A lysate prepared from a wild-type strain expressing endogenously tagged Bet5-HA3 was run through a Superose 6 column and the elution profile was subsequently monitored by anti-HA western blotting of fractions. Levels of the protein in any given fraction are represented as percentage of the total signal in the column. The elution profile was smoothened using the ‘Simple Spline Curve’ option of SigmaPlot’s Graph menu. Elution positions of protein standards (in kDa) are indicated on the top. (PDF) [file pgen.1008557.s003.pdf]

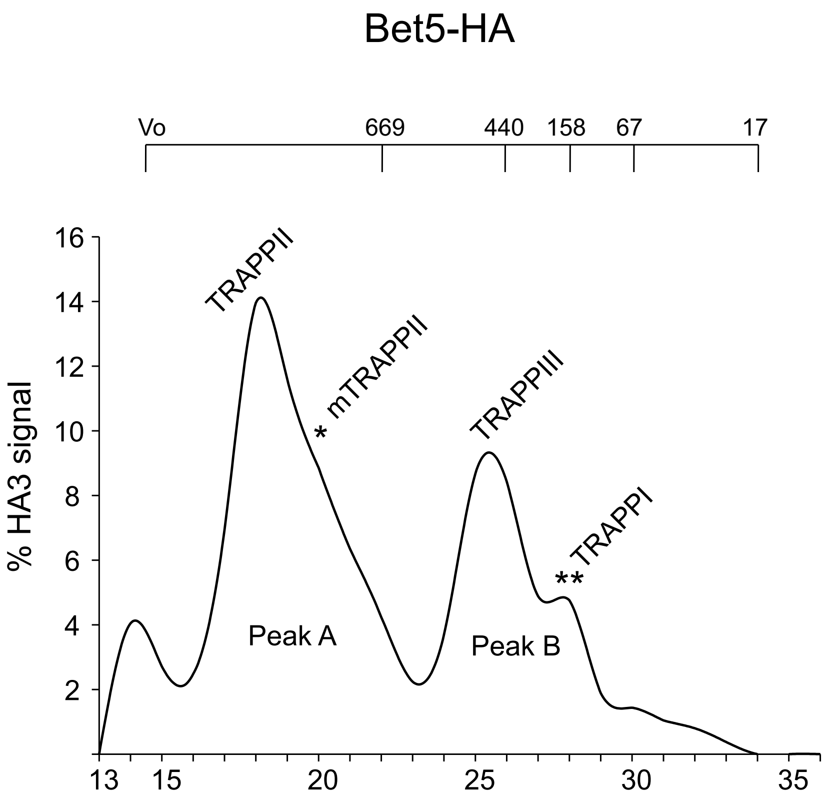

S3 Fig. Gel filtration analysis of Bet5-HA3 TRAPPs

A lysate prepared from a wild-type strain expressing endogenously tagged Bet5-HA3 was run through a Superose 6 column and the elution profile was subsequently monitored by anti-HA western blotting of fractions. Levels of the protein in any given fraction are represented as percentage of the total signal in the column. The elution profile was smoothened using the 'Simple Spline Curve' option of SigmaPlot's Graph menu.Elution positions of protein standards (in kDa) are indicated on the top.
