# Supplementary material for: Characterization of Aspergillus nidulans TRAPPs uncovers unprecedented similarities between fungi and metazoans and reveals the modular assembly of TRAPPII
Source: PLoS Genet. 2019 Dec 23;15(12):e1008557. doi: 10.1371/journal.pgen.1008557 (PMC6946167; doi:10.1371/journal.pgen.1008557)
Supplement: S4 Fig — Parallel S-agarose affinity purifications of TRAPP complexes from wild-type and indicated mutant cells were carried out using Trs120-S (TRAPPII) and Bet5-S (all TRAPPs). Proteins eluted from the S-agarose resin were analyzed by shotgun MS/MS. For each mutant condition, the PSM scores obtained as in Fig 5 were plotted as bar diagrams relative to the corresponding scores in the wild-type, which were set as 100%. Diagrams on the right represent the schematic composition of TRAPP complexes. (A) Bet5-S and Trs120-S in trs33Δ cells. (Note that this plots are the same as those displayed in Fig 5B, and are additionally shown here for convenience, to facilitate comparison with those shown in panels B and C.) (B) Bet5-S and Trs120-S in trs65Δ cells. (C) Bet5-S and Trs120-S in trs33Δ trs65Δ cells rescued with rab1* and rab11*. (PDF) [file pgen.1008557.s004.pdf]

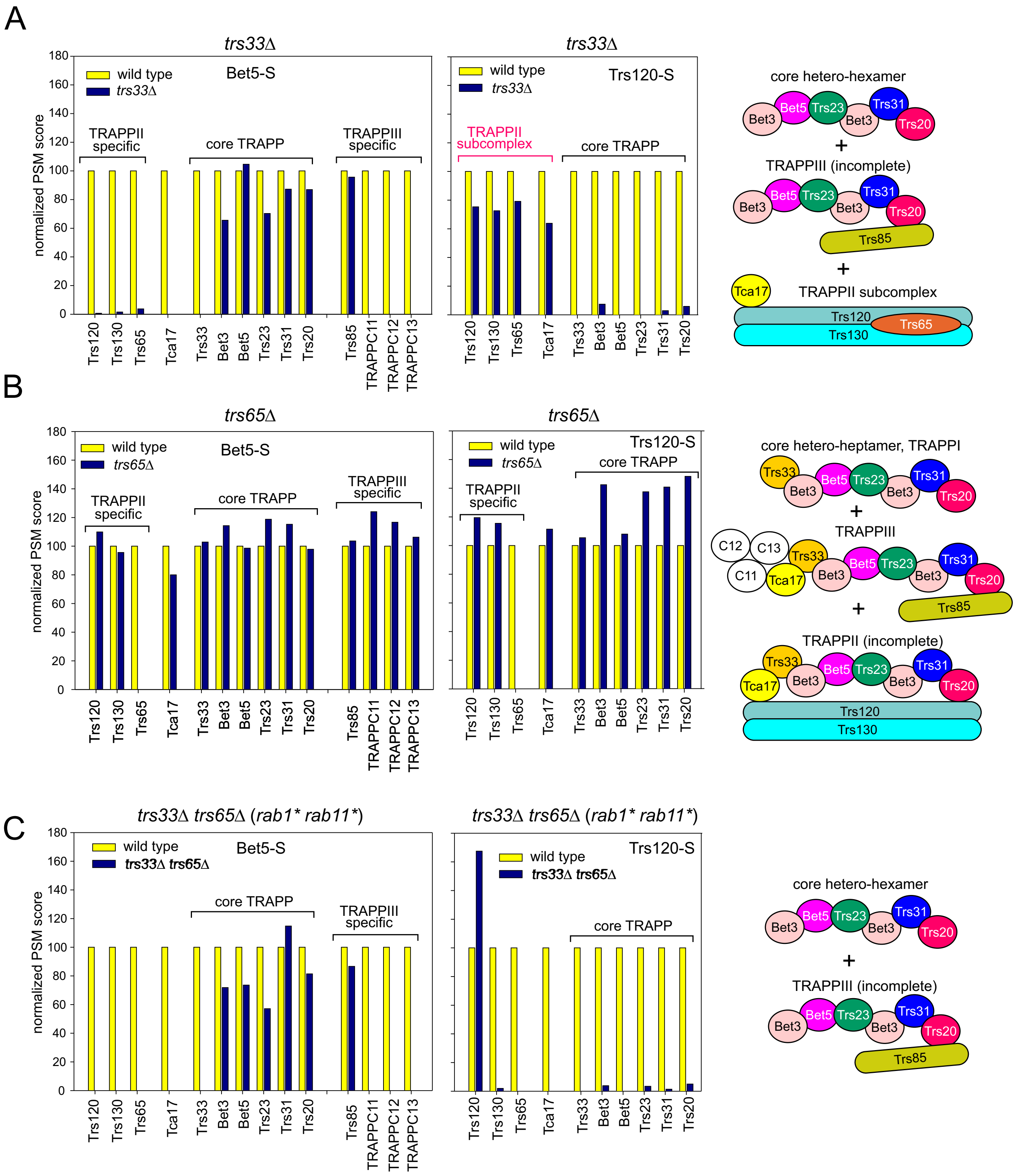

S4 Fig. MS/MS shotgun analyses of *trs33*Δ, *trs65*Δ and the double mutant strains.
Parallel S-agarose affinity purifications of TRAPP complexes from wild-type and indicated mutant cells were carried out using Trs120-S (TRAPPII) and Bet5-S (all TRAPPs). Proteins eluted from the S-agarose resin were analyzed by shotgun MS/MS. For each mutant condition, the PSM scores obtained as in Fig 5 were plotted as bar diagrams relative to the corresponding scores in the wild-type, which were set as 100%. Diagrams on the right represent the schematic composition of TRAPP complexes. (A) Bet5-S and Trs120-S in *trs33*Δ cells. (Note that this plots are the same as those displayed in Fig. 5B, and are additionally shown here for convenience, to facilitate comparison with those shown in panels B and C.) (B) Bet5-S and Trs120-S in *trs65*Δ cells. (C) Bet5-S and Trs120-S in *trs33*Δ *trs65*Δ cells rescued with *rab1** and *rab11**.
