# Supplementary material for: Characterization of Aspergillus nidulans TRAPPs uncovers unprecedented similarities between fungi and metazoans and reveals the modular assembly of TRAPPII
Source: PLoS Genet. 2019 Dec 23;15(12):e1008557. doi: 10.1371/journal.pgen.1008557 (PMC6946167; doi:10.1371/journal.pgen.1008557)
Supplement: S5 Fig — Wild-type and trs33Δ cells expressing Trs120-GFP. The images (shown in both colour and inverted greyscale for clarity) are maximal intensity projections. The graph on the right shows the quantitation of the Trs120-GFP fluorescence signal in TGN cisternae of N = 13 wild-type and N = 12 in trs33Δ cells, respectively. The two datasets differ significantly as determined by an unpaired t-test. (PDF) [file pgen.1008557.s005.pdf]

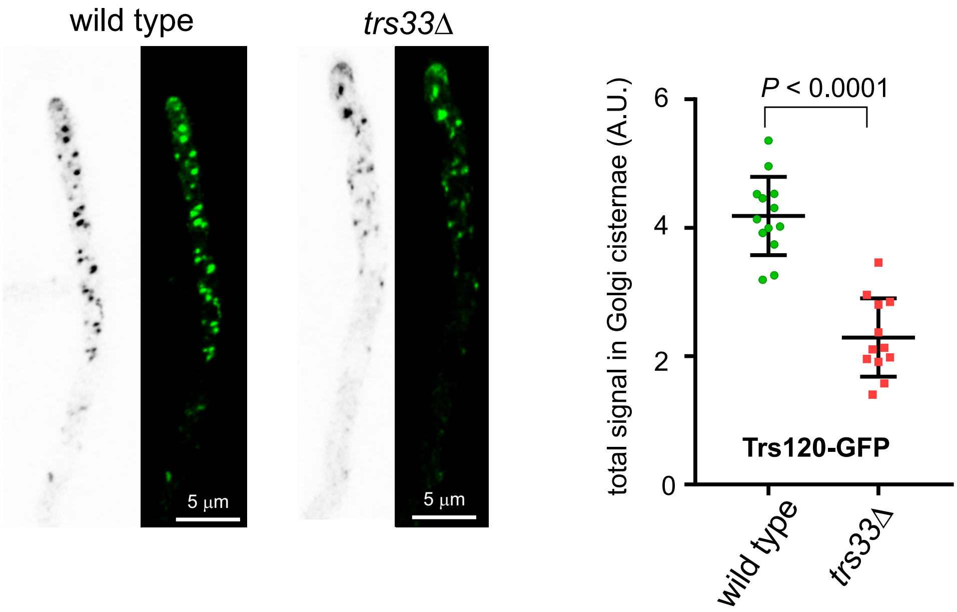

S5 Fig. *trs33*Δ partially delocalizes Trs120

Wild-type and *trs33*Δ cells expressing Trs120-GFP. The images (shown in both colour and inverted greyscale for clarity) are maximal intensity projections. The graph on the right shows the quantitation of the Trs120-GFP fluorescence signal in TGN cisternae of N = 13 wild-type and N = 12 in *trs33*Δ cells, respectively. The two datasets differ significantly as determined by an unpaired *t*-test.
