# Supplementary material for: Characterization of Aspergillus nidulans TRAPPs uncovers unprecedented similarities between fungi and metazoans and reveals the modular assembly of TRAPPII
Source: PLoS Genet. 2019 Dec 23;15(12):e1008557. doi: 10.1371/journal.pgen.1008557 (PMC6946167; doi:10.1371/journal.pgen.1008557)
Supplement: S6 Fig — The elution profiles on Superose 6 of TRAPPs monitored with Tca17-HA3 are shown. The experiments were carried out using 300 mM or 600 mM KCl to illustrate the fact that TRAPPIII containing Tca17 becomes detectable if the salt concentration is reduced, indicating that Tca17 is loosely associated to the complex. Elution profiles were smoothened using the ‘Simple Spline Curve’ option of SigmaPlot’s Graph menu. (PDF) [file pgen.1008557.s006.pdf]

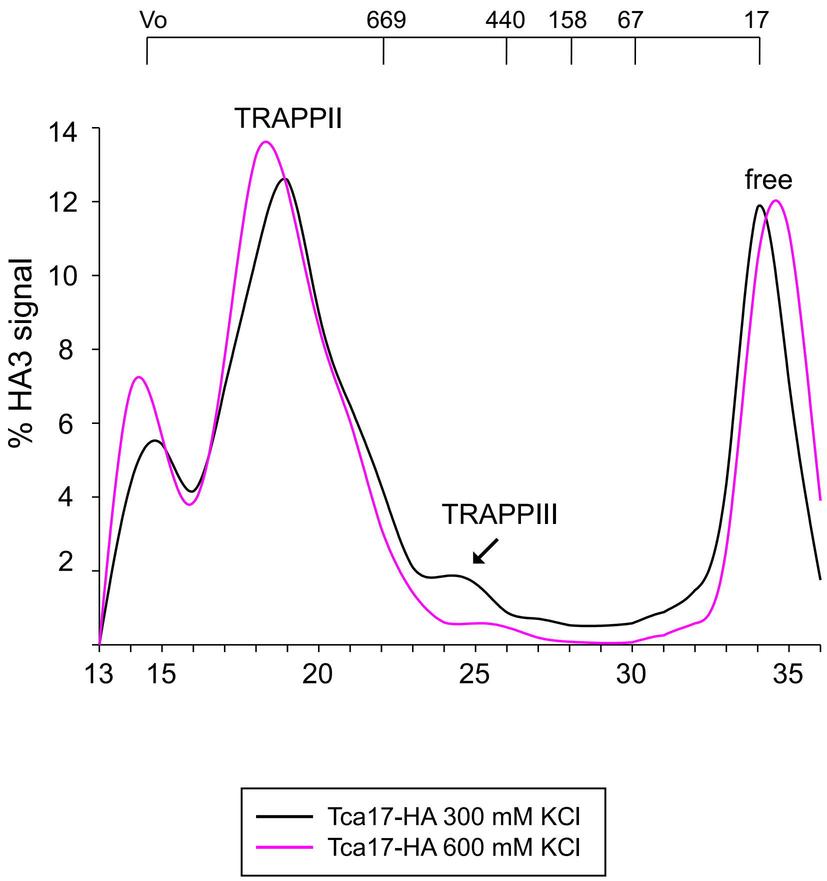


**S6 Fig. Tca17-containing TRAPPs at different KCl concentrations**

The elution profiles on Superose 6 of TRAPPs monitored with Tca17-HA3 are shown. The experiments were carried out using 300 mM or 600 mM KCl to illustrate the fact that TRAPPIII containing Tca17 becomes detectable if the salt concentration is reduced, indicating that Tca17 is loosely associated to the complex. Elution profiles were smoothened using the 'Simple Spline Curve' option of SigmaPlot's Graph menu.
