# Supplementary material for: Characterization of Aspergillus nidulans TRAPPs uncovers unprecedented similarities between fungi and metazoans and reveals the modular assembly of TRAPPII
Source: PLoS Genet. 2019 Dec 23;15(12):e1008557. doi: 10.1371/journal.pgen.1008557 (PMC6946167; doi:10.1371/journal.pgen.1008557)
Supplement: S7 Fig — The composition of TRAPP complexes copurifying with Tca17-S in an affinity column was determined by shotgun sequencing. Note that all TRAPPIII-specific proteins including Trs85 copurify with Tca17. The scheme is an interpretation of the complexes containing Tca17. (PDF) [file pgen.1008557.s007.pdf]

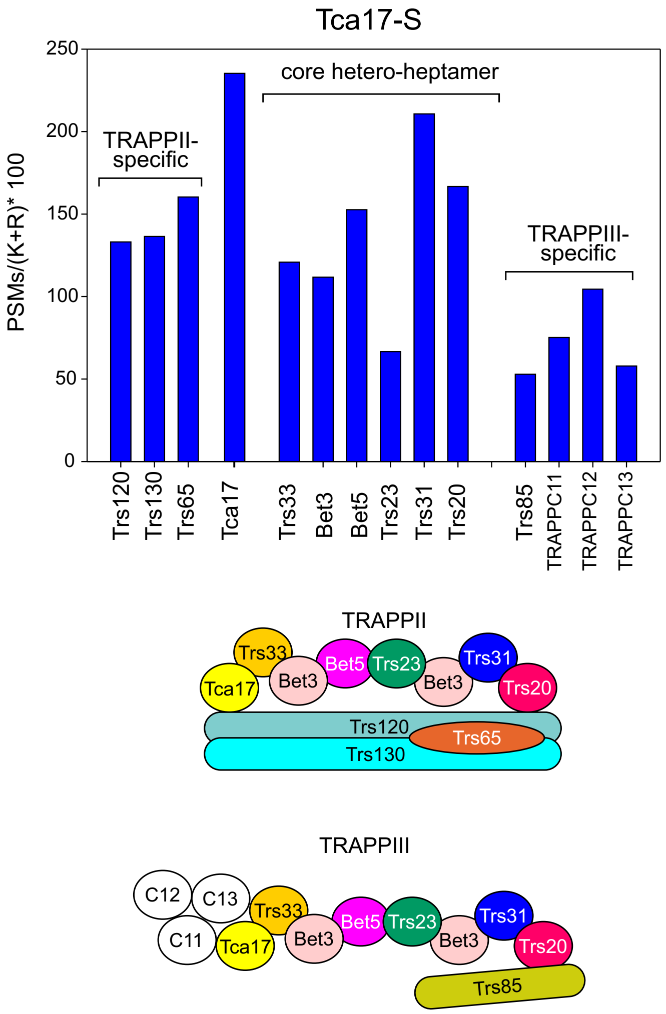

S7 Fig. MS/MS shotgun of Tca17-containing TRAPP complexes
The composition of TRAPP complexes copurifying with Tca17-S in an affinity column was determined by shotgun sequencing. Note that all TRAPPIII-specific proteins including Trs85 copurify with Tca17. The scheme is an interpretation of the complexes containing Tca17.
