# Supplementary material for: Characterization of Aspergillus nidulans TRAPPs uncovers unprecedented similarities between fungi and metazoans and reveals the modular assembly of TRAPPII
Source: PLoS Genet. 2019 Dec 23;15(12):e1008557. doi: 10.1371/journal.pgen.1008557 (PMC6946167; doi:10.1371/journal.pgen.1008557)
Supplement: S1 Table — (PDF) [file pgen.1008557.s008.pdf]

Table S1, strains used in this work

Strain code	Genotype
MAD3921	trs120::gfp::pyrG^{Af} pyrG89; pyroA4 nkuAΔ::bar
MAD4919	hypA1; wA3; inoB2 nkuAΔ::bar; rabE^{D125E}
MAD4963	pyrG89; bet5::ha$_3$::pyrG^{Af}; pyroA4 nkuAΔ::bar
MAD5319	pyrG89; pyroA4 nkuAΔ::bar; riboB2
MAD5321	pyroA4 nkuAΔ::bar; riboB2
MAD5328	pyrG89; pyroA4 nkuAΔ::bar; trs65::S-tag::pyrG^{Af}
MAD5365	trs120::S-tag::pyrGAf pabaA1 pyrG89?; nkuAΔ::bar?; trs130::ha$_3$::pyrG^{Af}
MAD5696	pyrG89; pyroA4 nkuAΔ::bar; trs65Δ::pyrG^{Af} riboB2
MAD5718	trs33Δ::riboB^{Af}; pyroA4 nkuAΔ::bar; riboB2
MAD5728	pyrG89; wA3; pyroA4 nkuAΔ::bar; rab11^{D125E}
MAD5736	pyrG89; pyroA4 nkuAΔ::bar
MAD5957	pyrG89?; wA3; rab11^{D125E}; inoB2 nkuAΔ::bar; trs65Δ::pyrg^{Af} riboB2
MAD5983	pyrG89? trs33Δ::riboB^{Af}; wA3; inoB2 nkuAΔ::bar; rab11^{D125E} trs65Δ::pyrg^{Af} riboB2
MAD6101	pyrG89; rab1$^{D124E:}$:riboB^{Af}; pyroA4 nkuAΔ::bar; riboB2
MAD6160	pyrG89; rab1$^{D124E:}$:riboB$^{Af;}$ pyroA4 nkuAΔ::bar; rab11^{D125E} riboB2?
MAD6284	pyrG89; rab1$^{D124E:}$:riboB$^{Af;}$ pyroA4 nkuAΔ::bar; bet3Δ::pyrG^{Af}; rab11^{D125E} riboB2?
MAD6286	pyrG89; bet5Δ::pyrG^{Af}; rab1$^{D124E:}$:riboB$^{Af;}$ pyroA4 nkuAΔ::bar; rab11^{D125E} riboB2?
MAD6292	pyrG89 trs20Δ::pyrG$^{Af;}$ rab1^{D124E}::riboB$^{Af;}$ pyroA4 nkuAΔ::bar; rab11^{D125E} riboB2?
MAD6294	trs31Δ::pyrG^{Af} pyrG89; rab1$^{D124E:}$:riboB^{Af}; pyroA4 nkuAΔ::bar; rab11^{D125E} riboB2?
MAD6298	trs20Δ::pyrG^{Af} pyrG89; wA3; pyroA4 nkuAΔ::bar; rab11^{D125E}
MAD6303	pyrG89; rab1$^{D124E:}$:riboB$^{Af;}$ trs23Δ::pyrG^{Af} pyroA4 nkuAΔ::bar; rab11^{D125E} riboB2?
MAD6347	pyrG89; rab1$^{D124E:}$:pyroA^{af}; pyroA4? inoB2 nkuAΔ::bar; trs65Δ::pyrG^{Af} rab11^{D125E} riboB2
MAD6354	pyrG89; rab1^{D124E}::pyroA^{Af}; pyroA4 nkuAΔ::bar; trs65Δ::pyrG^{Af} riboB2
MAD6360	trs33Δ::pyrG^{Af} pyrG89; wA3; pyroA4 nkuAΔ::bar; rab11^{D125E}
MAD6367	trs33Δ::pyrG^{Af} pyrG89; rab1^{D124E}::riboB$^{Af;}$ pyroA4 nkuAΔ::bar; riboB2
MAD6369	trs33Δ::pyrG^{Af} pyrG89; rab1^{D124E}::riboB$^{Af;}$ rab11^{D125E}; pyroA4 nkuAΔ::bar; riboB2?
MAD6377	trs33Δ::riboB^{Af} pyrG89; rab1$^{D124E:}$:pyroA$^{Af;}$ pyroA4? inoB2 nkuAΔ::bar; rab11^{D125E} trs65Δ::pyrG^{Af} riboB2
MAD6385	pyrG89 trs120Δ::pyrG^{Af}; wA3; pyroA4 nkuAΔ::bar; rab11^{D125E}
MAD6387	pyrG89; wA3; pyroA4 nkuAΔ::bar; trs130Δ::pyrG^{Af} rab11^{D125E}
MAD6389	pyrG89 trs120Δ::pyrG^{Af}; rab1^{D124E}::riboB$^{Af;}$ pyroA4 nkuAΔ::bar; rab11^{D125E} riboB2?
MAD6391	pyrG89; rab1$^{D124E:}$:riboB^{Af}; pyroA4 nkuAΔ::bar; trs130Δ::pyrG^{Af} rab11^{D125E} riboB2?
MAD6461	pyrG89?; trs85Δ::pyrG^{Af} nkuΔ::bar?; riboB2
MAD6512	pyrG89 pabaA1; nkuAΔ::bar
MAD6526	pabaA1; nkuAΔ::bar; riboB2
MAD6573	trs20::S-tag::pyrG^{Af} pabaA1 pyrG89; nkuAΔ::bar
MAD6575	trs20::ha$_3$::pyrG^{Af} pabaA1 pyrG89; nkuAΔ::bar
MAD6577	pabaA1 pyrG89; trs85-S-tag::pyrG^{Af} nkuAΔ::bar
MAD6600	pyrG89; trs23::ha$_3$::pyrG^{Af} nkuAΔ::bar pyroA4; riboB2
MAD6606	trs33Δ::riboB^{Af} trs120::gfp::pyrG^{Af} pyrG89?; pyroA4 nkuAΔ::bar?; riboB2?
MAD6619	trs120::S-tag::pyrG^{Af} pyrG89?; wA3; nkuAΔ::bar?; trs130::ha$_3$::pyrG^{Af} rab11^{D125E}
MAD6624	trs120::S-tag::pyrG^{Af} trs20Δ::pyrG^{Af} pabaA1 pyrG89?; wA3; pyroA4 nkuAΔ::bar?; rab11^{D125E} trs130::ha$_3$::pyrG^{Af}
MAD6647	trs120::ha$_3$::pyrG^{Af} pyrG89?; bet5::S-tag::pyrG^{Af}; nkuAΔ::bar?; rab11^{D125E} trs130::ha$_3$::pyrG^{Af}
MAD6650	trs20Δ::pyrG^{Af} trs120::ha$_3$::pyrG^{Af} pyrG89?; bet5::S-tag::pyrG$^{Af;}$ pyroA4 nkuAΔ::bar?; rab11^{D125E} trs130::ha$_3$::pyrG^{Af}
MAD6652	pabaA1 pyrG89?; bet5::S-tag::pyrG^{Af}; nkuAΔ::bar?; trs130::ha$_3$::pyrG^{Af}
MAD6710	trs120::S-tag::pyrG^{Af} pabaA1 pyrG89?; rab1^{D124E}::riboB^{Af}; pyroA4 nkuAΔ::bar?; rab11^{D125E} trs130::ha$_3$::pyrG^{Af}
MAD6711	trs120::S-tag::pyrG^{Af} trs31Δ::pyrG^{Af} pabaA1 pyrG89?; rab1$^{D124E:}$:riboB^{Af}; nkuAΔ::bar?; rab11^{D125E} trs130::ha$_3$::pyrG^{Af}
MAD6716	trs120::S-tag::pyrG^{Af} pabaA1 pyrG89?; rab1$^{D124E:}$:riboB^{Af}; trs23Δ::pyrG^{Af} nkuAΔ::bar?; rab11^{D125E} trs130::ha$_3$::pyrG^{Af}

MAD6745	trs31Δ::pyrGAf pyrG89?; rab1^{D124E}::riboB^{Af} ; bet5::S-tag::pyrG^{Af}; pyroA4 nkuAΔ::bar?; trs130-ha$_3$::pyrG^{Af} rab11^{D125E}
MAD6746	pyrG89?; rab1^{D124E}::riboB^{Af}; bet5::S-tag::pyrG^{Af}; trs23Δ::pyrG^{Af} pyroA4 nkuAΔ::bar?; trs130::ha$_3$::pyrG^{Af} rab11^{D125E}
MAD6752	trs33Δ::riboB^{Af} pyrG89?; bet5::S-tag::pyrG^{Af}; nkuAΔ::bar?; trs130::ha$_3$::pyrG^{Af} rab11^{D125E} trs65Δ::pyrg^{Af} riboB2?
MAD6753	pyrG89; rab1^{D124E}::riboB^{Af}; tca17Δ::pyrg^{Af}; pyroA4 nkuAΔ::bar; riboB2? rab11^{D125E}
MAD6757	trs33Δ::riboB^{Af} trs120::S-tag::pyrG^{Af} pabaA1 pyrG89?; nkuAΔ::bar?; rab11^{D125E} trs65Δ::pyrg^{Af} trs130::ha$_3$::pyrG^{Af}
MAD6758	pyrG89; wA3; tca17Δ::pyrg^{Af}; pyroA4 nkuAΔ::bar; rab11^{D125E}
MAD6778	pabaA1 pyrG89; nkuAΔ::bar; tca17::S-tag::pyrG^{Af}
MAD6856	trs33Δ::riboB^{Af} trs120::S-tag::pyrG^{Af} pabaA1 pyrG89?; pyroA4 nkuAΔ::bar?; trs130::ha$_3$::pyrG^{Af} riboB2?
MAD6863	trs33Δ::riboB^{Af} pyrG89?; bet5::S-tag::pyrG^{Af}; pyroA4 nkuAΔ::bar?; trs130::ha$_3$::pyrG^{Af} riboB2?
MAD6916	trs120::S-tag::pyrG^{Af} pabaA1 pyrG89?; wA3; tca17Δ::pyrg^{Af}; nkuAΔ::bar?; rab11^{D125E} trs130::ha$_3$::pyrG^{Af}
MAD6919	pabaA1 pyrG89?; bet5::S-tag::pyrG^{Af}; nkuAΔ::bar?; trs130::ha$_3$::pyrG^{Af} trs65Δ::pyrG^{Af} riboB2
MAD6921	trs120::S-tag::pyrG^{Af} pabaA1 pyrG89?; nkuAΔ::bar?; trs65Δ::pyrG^{Af} trs130::ha$_3$::pyrG^{Af}
MAD6952	pyrG89; wA3 tca17Δ::pyrg^{Af}; bet5::S-tag::pyroA^{Af}; pyroA4 nkuAΔ::bar; rab11^{D125E}
MAD6989	trs120Δ::pyrG^{Af} pyrG89; pyroA4 nkuAΔ::bar; rab11^{D125E} trs65::S-tag::pyroA^{Af}
MAD7005	pyrG89 pabaA1; trs85::ha$_3$::pyrG^{Af} nkuAΔ::bar
MAD7015	trs120Δ::pyrG^{Af} pyrG89?; bet5::S-tag::pyrG^{Af}; nkuAΔ::bar?; rab11^{D125E} trs130::ha$_3$::pyrG^{Af}
MAD7034	trs120::S-tag::pyrG^{Af} pabaA1 pyrG89?; trs23::ha$_3$::pyrG^{Af} pyroA4 nkuAΔ::bar?; riboB2
MAD7036	pyrG89 pabaA1; trs85::S-tag::pyrG^{Af} trs23::ha$_3$::pyrG^{Af} pyroA4 nkuAΔ::bar; riboB2
MAD7038	trs120Δ::pyrG^{Af} pyrG89?; bet5::S-tag::pyrG^{Af}; pyroA4 nkuAΔ::bar? trs23::ha$_3$::pyrG^{Af}; rab11^{D125E} riboB2
MAD7046	pyrG89; pyroA4 nkuAΔ::bar; tca17::ha3::pyrG^{Af}
MAD7049	trs85Δ::pyrG^{Af} bet5::S-tag::pyrG^{Af} trs23::ha3::pyrG^{Af} pabaA1 pyrG89? nkuAΔ::bar?
MAD7053	trs85Δ::pyrG^{Af} trs120Δ::pyrG^{Af} rab11^{D125E} bet5::S-tag::pyrG^{Af} trs23::ha$_3$::pyrG^{Af} pyroA4 riboB2 pyrG89? nkuAΔ::bar?
MAD7088	trs33Δ::pyrG^{Af} pyrG89?; trs23::ha$_3$::pyrG^{Af} pyroA4 nkuAΔ::bar?
